# A One Health Scoping Review of Antimicrobial Resistance in Cambodia: Evidence Mapping and Insights into *Klebsiella pneumoniae*

**DOI:** 10.3390/microorganisms14102161

**Published:** 2026-09-24

**Authors:** Sao Puth, Chanroth Chhoung, Limmuy Hav, Chandy Rin, Kennarey Seang, Luis Sagaon-Teyssier, Vonthanak Saphonn

**Affiliations:** 1 The Global Health Research Center (GHRC), University of Health Sciences (UHS), 73 Preah Monivong Blvd, Sangkat Sras Chok, Khan Daun Penh, Phnom Penh 12000, Cambodia; 2 Aix Marseille University, Inserm, IRD, SESSTIM, Sciences Economiques & Sociales de la Santé & Traitement de l’Information Médicale, ISSPAM, 13005 Marseille, France

**Keywords:** antimicrobial resistance, *Klebsiella pneumoniae*, CRKP, ESBL, Cambodia, One Health

## Abstract

Antimicrobial resistance (AMR) is a growing global public health threat that disproportionately affects low- and middle-income countries (LMICs), including Cambodia. The distribution of AMR across interconnected human, animal, food, and environmental sectors highlights the importance of using a One Health approach to better understand and address this issue. In Cambodia, AMR is increasingly reported among key pathogens, including *K. pneumoniae*. In particular, the emergence of colistin and carbapenem resistance in this organism is of major clinical concern, as it compromises the effectiveness of critical antimicrobial agents used for the treatment of severe multidrug-resistant (MDR) infections, leading to increased morbidity, mortality, and healthcare costs. This scoping review aims to systematically map and summarize available One Health evidence on AMR in Cambodia across human, animal, food, and environmental sectors. By integrating evidence from these interconnected sectors, the review identifies major AMR patterns, potential transmission pathways and associated risk factors, as well as priority pathogens to inform future research priorities and policy development. Following the PRISMA-ScR guidelines, we searched PubMed/MEDLINE, Europe PMC, Google Scholar, Google Search, and grey literature sources. Of the 611 records identified, 455 unique records were screened, resulting in 94 studies included in the final analysis. Data were summarized narratively by categorizing evidence across human, animal, food, and environmental sectors. *K. pneumoniae* was included as a pre-specified pathogen of interest, because of its clinical relevance and AMR relevance in the Cambodian context. AMR burden in Cambodia is high, particularly among Gram-negative bacteria such as *K. pneumoniae*. The included studies reported substantial AMR, with estimates varying considerably according to population, setting, specimen type, study period, and laboratory definitions. High rates of extended-spectrum β-lactamase (ESBL) production and reported colistin and carbapenem resistance were observed in both hospital and community settings. *K. pneumoniae* is a key pathogen in healthcare-associated infections and neonatal sepsis, with frequent reports of multidrug-resistant (MDR) and carbapenem-resistant (CRKP) strains. Evidence linking human, animal, food, and environmental sectors was limited. As the data on shared resistance determinants and genetically similar isolates were cross-sectional in nature; they only suggest potential pathways and cannot prove directional transmission. Major drivers of AMR and associated factors include inappropriate antimicrobial use, agricultural practices, limited diagnostic capacity, inadequate infection prevention and control, and environmental contamination. Significant gaps remain in genomic surveillance and cross-sectoral research on AMR in Cambodia. Addressing these gaps will require strengthening One Health efforts and integrated cross-sectoral surveillance. *K. pneumoniae* may serve as a One Health sentinel pathogen for healthcare-related AMR surveillance, particularly ESBL and CRKP strains. However, this potential needs to be evaluated through pathogen-neutral, longitudinal, and cross-sectoral surveillance. Strengthening laboratory capacity, improving antimicrobial stewardship, and developing integrated surveillance systems are critical to mitigating AMR in the country.

## 1. Introduction

Antimicrobial resistance (AMR) has become one of the most serious challenges to global health. Compromising the effectiveness of many widely used antibiotics, the consequences of this phenomenon include greater infection-related mortality, and dramatic increases in health care costs. The burden of AMR is greatest in low- and middle-income countries, where diverse issues including fragile healthcare systems, insufficient antimicrobial stewardship, and environmental pressures on infection control have led to the rapid emergence and spread of resistant pathogens [1,2,3,4,5,6,7]. In 2019, AMR was associated with an estimated 4.95 million deaths globally, including 1.27 million deaths directly attributable to resistant infections. Without effective interventions, the annual number of deaths attributable to AMR could reach 10 million by 2050 [8].

Cambodia is an important setting for understanding AMR dynamics in Southeast Asia because of its rapidly changing healthcare system, substantial agricultural activities, and the presence of diverse types of environmental interfaces [9,10,11,12,13,14,15,16,17,18]. In healthcare settings, the emergence and amplification of resistant strains is partially the result of inappropriate prescription of antibiotics [4,6,7,19,20,21,22,23,24], self-medication practices, widespread over-the-counter access to antibiotics without adequate clinical oversight [4,6,7,25], prolonged hospitalization, inadequate management of infections, limited diagnostic capacity, weak microbiological surveillance systems, and the circulation of substandard or falsified medicines [2,3,5]. In agriculture, intensive household livestock rearing and informal animal markets contribute to AMR [9,10,11,12,18,25,26,27,28], as does antimicrobial use in livestock and aquaculture. Environmental exposures, including contaminated food, water and soil, as well as inadequate water, sanitation and hygiene (WASH) systems, also contribute to the persistence and dissemination of resistant organisms [12,29,30,31,32,33]. These multi-sectoral interactions highlight the importance of strengthening Cambodia’s nascent One Health coordination to improve understanding of the factors contributing to AMR emergence and dissemination across human, animal, food, and environmental sectors [9,10,11,12,18,28,34].

AMR in Cambodia is characterized by a great number of resistances among key bacterial pathogens, particularly those belonging to the Enterobacterales family, including *Klebsiella pneumoniae* [20,21,22,24,25,27,35,36,37,38,39,40,41,42,43,44,45]. The latter is one of six pathogens in the World Health Organization’s ESKAPE list (*Enterococcus faecium*, *Staphylococcus aureus*, *Klebsiella pneumoniae*, *Acinetobacter baumannii*, *Pseudomonas aeruginosa* and *Enterobacter* spp.), which identifies priority organisms for surveillance and intervention. *K. pneumoniae* is included in the list because of its high capacity to acquire and disseminate resistance determinants, including plasmid-mediated extended-spectrum beta-lactamases (ESBLs) which lead to the development of multi-drug resistance (MDR) profiles [10,11,12,22,24,25,40,41,42]. This is especially the case for last-line (also called last-resort) antibiotics, such as colistin and carbapenems [10,11,12,18,24,46,47,48,49]. In Cambodia, *K. pneumoniae* shows a high prevalence of carbapenem resistance, highlighting the organism’s importance as a priority pathogen for AMR surveillance [50]. Moreover, monitoring *K. pneumoniae*’s resistance could act as a proxy for monitoring trends in AMR in Cambodia more generally [20,22,23,24,27,51], and provide important data on regional and global AMR growth.

Unlike several other clinically important AMR pathogens, most Cambodian evidence on *K. pneumoniae* originates from healthcare settings [28,29,30,31,32,33,34,45,52,53,54,55,56,57,58,59,60,61,62,63,64]. Potential transmission or dissemination pathways identified in the literature include person-to-person contact (e.g., health workers’ hands), contaminated medical devices, and persistence or dissemination within healthcare environments [22,24,27,51]. Genomic and epidemiological studies also indicate that *K. pneumoniae* can occur beyond healthcare settings, including in community and animal populations; however, the characterization of the direction, timing, and extent of transmission between these compartments remains incomplete [11,20,23,38,39].

Despite a growing body of literature, research on AMR in Cambodia is fragmented across research disciplines and sectors. Consolidating available evidence is therefore crucial to establish a baseline for future research and policy [9,10,11,12,18]. Recent reports describe efforts to strengthen One Health coordination in Cambodia [65], while the country has developed a multisectoral national action plan on AMR for 2025–2029 [66]. These developments suggest the need for evidence that can characterize AMR patterns across human, animal, food, and environmental sectors.

In this context, the primary aim of this scoping review was to map the One Health evidence produced to date on AMR in Cambodia, including its geographical distribution, key findings, and research gaps. More specifically, we aimed to characterize the extent to which evidence on AMR in Cambodia addresses human, animal, food, and environmental sectors, whether explicitly or indirectly, within a One Health framework.

The review also aimed to identify major drivers and risk factors associated with AMR in *K. pneumoniae* and to assess the relevance of focusing on this organism as a potential sentinel for healthcare-associated AMR surveillance within a One Health framework.

## 2. Materials and Methods

### 2.1. Study Design and Search Strategy

Using the PRISMA-ScR methodology [67], the present scoping review mapped evidence on AMR in Cambodia across human, animal, food, and environmental sectors, with a pre-specified focus on *K. pneumoniae*. We searched PubMed/MEDLINE, Europe PMC, Google Scholar, and Google Search. The latter two sources were used as supplementary discovery sources because relevant Cambodian evidence is not consistently indexed in bibliographic databases. We also searched targeted government and institutional sources for relevant grey literature, as another supplementary discovery source. The search was limited to studies conducted in Cambodia or among Cambodian populations and used combinations of controlled vocabulary and free-text terms relating to AMR, resistance phenotypes, bacterial pathogens, *K. pneumoniae*, and relevant human, animal, food, and environmental contexts. Database-specific search concepts and Boolean strategies and the research results are provided in Supplementary Table S1. Literature published through 11 May 2026 was eligible. The review protocol was not prospectively registered in a public registry or protocol repository.

### 2.2. Study Selection Process

Records were retained from PubMed/MEDLINE, Europe PMC, Google Scholar, and Google Search. They were pooled and imported into reference management software (ReadCube: https://www.readcube.com/), where duplicates were removed. The remaining unique records were then imported into Covidence (https://www.covidence.org/) for title/abstract and full-text screening. Three reviewers independently screened records for inclusion, with disagreements resolved through discussion and consensus.

The inclusion criteria were as follows: (i) study conducted in Cambodia or among Cambodian populations, involving human, animal, food, and/or environmental populations; (ii) study addressed AMR, including MDR, XDR, PDR, ESBL production, carbapenem resistance, or related resistance phenotypes; (iii) bacterial pathogens, particularly ESKAPE organisms and other Enterobacterales; (iv) epidemiological, clinical, laboratory, surveillance, or risk-factor outcomes relevant to AMR and One Health; (v) observational, surveillance, laboratory, or public-health study design; and (vi) publication in English without restriction by year. Only English-language publications were included, as the review team did not have sufficient translation capacity to consistently assess non-English records in a rigorous manner. Supplementary Table S2 details the inclusion and exclusion criteria.

### 2.3. Data Extraction and Summary

Essential information was independently extracted by three reviewers using a predefined Microsoft Excel template, including title, authors, publication date, study setting, population, sector, pathogen, AMR phenotype or determinant, key quantitative findings, and evidence relevant to risk factors or potential transmission pathways. Data were analyzed descriptively using narrative synthesis. Findings were organized to map AMR evidence across human, animal, food, and environmental sectors and to assess the potential role of *K. pneumoniae* within this evidence base. Because the included studies varied substantially in terms of design, population, sampling frame, laboratory methods, and definitions of resistance, no quantitative pooling or weighting of prevalence estimates was performed. Moreover, as the scoping review’s primary objective was evidence mapping for AMR, we did not conduct any formal risk-of-bias assessment.

## 3. Results

### 3.1. Evidence Landscape: One Health Mapping

Six hundred and eleven records were initially retained (PubMed/MEDLINE (*n* = 40), Europe PMC (*n* = 197), Google Scholar (*n* = 319), and Google Search (*n* = 55). These were pooled and imported into reference management software (ReadCube: https://www.readcube.com/) and subsequently imported into Covidence (https://www.covidence.org/) for title/abstract and full-text screening, where, totally, 156 duplicates were removed. Of the 455 unique records retained at this point, 356 did not meet the inclusion criteria during title/abstract screening (Figure 1). The remaining 99 records underwent full-text assessment, 5 being secondarily excluded (2 PhD theses, 1 article focusing on antiviral resistance, 1 on malaria, and 1 on helminth infection). Finally, 94 studies were included for data extraction, summary, and analysis, comprising 92 peer-reviewed articles and 2 grey literature sources (WHO, *n* = 1; institutional report, *n* = 1). The included studies were published between May 2009 [43] and April 2026 [42].

A summary of the 94 studies included demonstrated evidence that AMR occurs across interconnected human, animal, food, and environmental sectors. This highlighted the need for a One Health approach to investigate the emergence, transmission, and control of resistance in Cambodia (Supplementary Table S3). The majority of the studies focused on Enterobacterales, particularly *E. coli* and *K. pneumoniae* [20,21,22,24,25,27,35,36,37,38,39,40,41,42,43,44,45,68]. The remaining studies used animal [46,47,48,49], food [9,26,34,46,58,69], and environmental samples [9,10,11,12,18]. Furthermore, studies on *E. coli* and *K. pneumoniae* also included specific populations, particularly neonates and children [19,20,23,35,39,42,44,70,71]. *K. pneumoniae* appeared to be a major cause of infections in health facilities, including bloodstream infections and intensive care unit (NICU) outbreaks; this is unsurprising given its prevalence in Cambodia. *E. coli* exhibited a broad ecological distribution spanning community settings, food chains, and environmental reservoirs. Both *K. pneumoniae* and *E. coli* emerged as the dominant Enterobacteral species, representing 32% (30/94) of the all the articles included (Supplementary Table S3). The review included studies of Enterobacterales across human, animal, food, and environmental sectors, providing evidence relevant to potential interfaces between these sectors [9,10,11,12,18,28,34]. However, these studies generally provided associations, shared determinants, or genetic similarities rather than definitive longitudinal evidence establishing transmission direction.

Concerning other pathogens, *Salmonella* spp. studies included human [45,61], animal [56,63], and food [34,56,58,63] data, as well as studies involving neonates and children [29,32,33,54,64,72,73]. The available evidence predominantly concerned *S. Typhi*, *S. Paratyphi A*, and non-typhoidal *Salmonella*. In contrast, *Shigella* spp., *Neisseria gonorrhoeae*, *Staphylococcus aureus* (including Methicillin-resistant *Staphylococcus aureus* (MRSA), *Streptococcus pneumoniae*, *Mycobacterium tuberculosis*, and *Helicobacter pylori*) were primarily investigated in human clinical settings, with limited representation in animal, food, or environmental settings [30,31,74]. A small number of studies (*n* = 9) investigated mixed Enterobacteral pathogens in cross-sectoral settings, specifically highlighting the interconnectedness of AMR transmission pathways across human–animal–food–environment interfaces. Others (*n* = 7) focused on healthcare-associated transmission and vulnerable populations, particularly neonates and children [9,10,11,12,18,28,34].

### 3.2. AMR Patterns Across Key Pathogens

The summary of Cambodian AMR evidence reveals a consistently high and expanding burden of AMR across multiple priority pathogens (Supplementary Table S4). Bacterial organisms show distinct AMR patterns observed across clinical and community settings. Specifically, enteric bacterial pathogens, such as *Salmonella* spp. [29,32,33,34,45,54,56,58,61,63,64,72,73] and *Shigella* spp. [30,31,74], exhibit high levels of MDR and increasing fluoroquinolone and third-generation cephalosporin resistance, driven by well-characterized chromosomal mutations and plasmid-mediated mechanisms. Similarly, *Neisseria gonorrhoeae* shows reduced susceptibility to extended-spectrum cephalosporins in populations living with sexually transmitted infections (STIs) [52,57,59,60], while *Staphylococcus aureus*, including MRSA, remains prevalent across hospital, community, and livestock-associated settings [28,53,62,75,76,77]. *Streptococcus pneumoniae* continues to demonstrate evolving resistance to β-lactams, particularly in pediatric populations following vaccine-driven serotype replacement [78,79]. For *Mycobacterium tuberculosis*, evidence indicates an overall low burden of MDR-TB but emerging fluoroquinolone resistance associated with *rpoB* and *gyrA* mutations [80,81]. *Helicobacter pylori* shows high MDR (57–76%), particularly metronidazole resistance, with clarithromycin resistance linked to 23S rRNA mutations [82,83]. *Clostridioides difficile* shows notable colonization (<24%) and emerging resistance patterns among hospitalized adults, although AMR gene data remain limited [84,85]. In contrast, Enterobacterales (*E. coli* and *K. pneumoniae*) show a substantially higher burden of AMR, with ESBL production reported across different populations and settings, ranging from 30–82%, and reaching up to 84% among community-carriage isolates and 98% among hospital-associated isolates [9,10,11,12,18,20,21,22,24,25,35,36,37,38,39,40,41,42,43,44,45,46,47,48]. Most of the resistance is driven by key determinants including *blaCTX-M*, *blaNDM*, *blaOXA-48*, and *mcr* genes (*mcr-1*, *mcr-3*, *mcr-8*), which are strongly associated with multidrug resistance (MDR). Although MDR Enterobacterales, especially *E. coli*, have been identified and extensively studied across the human, animal, food, and environmental sectors, most evidence on *K. pneumoniae* originates from hospital-based studies. This highlights major knowledge gaps regarding the occurrence, transmission, and resistance patterns of this pathogen in non-clinical settings.

The evolution of *K. pneumoniae* in Cambodia is particularly concerning because of its increasing resistance to last-resort antibiotics, including carbapenems and colistin, in certain settings. Carbapenem-resistant *K. pneumoniae* (CRKP), often associated with mobile genetic elements and high-risk clones, is strongly linked to healthcare-associated infections, including neonatal sepsis and bloodstream infections [9,10,11,12,18,20,21,22,24,25,35,36,37,38,39,40,41,42,43,44,45,46,47,48]. This finding supports the importance of healthcare-associated AMR surveillance using a One Health framework. It is important to highlight that as these studies used different designs and sampling methods, and relied entirely on cross-sectional data, this result cannot be interpreted as a national temporal trend.

### 3.3. Potential AMR Transmission Pathways in Cambodia

*K. Pneumoniae* has been primarily reported in healthcare-based settings; our summary of evidence from Cambodian studies on MDR Enterobacterales is based on findings from the human, animal, food, and environmental sectors (Supplementary Table S5). Collectively, these findings provide a broader understanding of AMR patterns across interconnected sectors, although integrated One Health investigations remain limited. ESBL-producing *E. coli*, especially those producing CTX-M enzymes, are potentially transmitted across human, livestock, and environmental reservoirs. Furthermore, studies have identified closely related clonal strains across these sectors within the same geographic regions, supporting the hypothesis of interconnection between sources. Specifically, high prevalence of ESBL-producing Enterobacterales in community settings (up to 84%) has been reported, with potential transmission strongly associated with exposure to fecally contaminated water and soil, highlighting the role of environmental contamination in sustaining and spreading resistant strains [10,11,18,41]. Similarly, MDR E. coli, including those producing mcr genes, are transmitted through the food chain and through direct contact with livestock. There is also strong evidence for the presence of mcr-1 and related resistance genes from animal products and food items on farms, as well as for high farm-level MDR prevalence [46,48,49]. This pattern suggests that antimicrobial use in agriculture may contribute not only to the dissemination but also to the selection and amplification of resistant organisms within the food animal production system, thereby facilitating their subsequent transmission to humans through the food chain.

In studies focusing on *Shigella* spp., potential transmission occurs mainly in community settings through person-to-person contact and through the contamination of water supplies, with poor sanitation systems leading to high rates of multidrug-resistant strains [30,31,74]. *S. Typhi* (particularly the multidrug-resistant H58 lineage) is strongly associated with waterborne transmission through fecal–oral routes, particularly affecting children; this highlights the critical role of water, sanitation, and hygiene systems in controlling enteric fever [29,32,33]. With regard to *S. aureus*, zoonotic transmission is the inferred pathway, with MRSA circulating amongst humans and livestock; a very small number of VRSA cases have been identified in dairy cattle, indicating the possible risk of occupational exposure [62,75,76]. The included articles also show a high prevalence of multidrug resistant *Salmonella* spp. strains (69 to 91%) circulating through livestock, slaughterhouses, and human populations, with contaminated food products potentially representing an important pathway for zoonotic transmission [56,58,63].

MDR and extensively drug-resistant (XDR) *N. gonorrhoeae* strains, including the FC428 lineage, have been identified through antimicrobial susceptibility and genomic surveillance studies. Their dissemination through international transmission networks contributes to the introduction and spread of resistant strains, influencing local resistance patterns [52,57,59]. Enterobacterial food-associated AMR is also significant with evident human transmission occurring predominantly through human consumption of contaminated fermented food items. Indeed, some of the included studies estimated that >50% of fermented items contained an MDR enterobacterial population [9,26,34]. In addition, aquatic habitats are a significant contributor to the MDR burden through the circulation of *Aeromonas* spp. and *Vibrio* spp. across aquaculture settings, fish markets, and human populations, a consequence of environmental contamination and the overuse of antibiotics in aquaculture [15,16].

At the genome level, horizontal gene transfer is an important mechanism of AMR transmission, where plasmid-mediated ESBL and other resistance determinants flow between human, animal, and environmental sectors, as well as through the transfer of large plasmids (e.g., megaplasmids) from one environment to another [10,11,86]. In community settings, persons living in close quarters or in common spaces drive transmission of ESBL-producing Enterobacterales and MRSA [25,28,39]. Neonatal infections are primarily transmitted through human-to-human hospital-based sources or environmental sources rather than mother-to-child transmission [87,88]. Inappropriate antibiotic use in human healthcare and livestock production remains a major driver of AMR emergence and dissemination. Widespread antibiotic misuse, combined with weak regulatory enforcement, promotes the selection and spread of resistant organisms and resistance determinants across interconnected human, animal, food, and environmental sectors [1,4,25,89].

It is important to recognize that healthcare settings can act as important reservoirs and transmission hubs for ESBL-producing Enterobacterales, facilitating the dissemination of resistant organisms into community settings [24,25,27]. *K. pneumoniae* is particularly noted for its ease of transmission in hospitals, especially in NICUs, where whole-genome sequencing has documented clear outbreak clusters (all of which are genetically very similar). Persistent detection of related strains suggests ongoing challenges in controlling transmission in hospital settings [20,23].

Moreover, the strong presence of ESBL-producing Enterobacterales across all sectors, combined with evidence of hospital-driven transmission involving genetically related *K. pneumoniae* clones, environmental persistence, and plasmid-mediated gene exchange, provides a biological and ecological framework that facilitates the emergence and dissemination of *K. pneumoniae*. In this context, the increasing detection of carbapenem-resistant *K. pneumoniae* does not represent an isolated phenomenon, but rather the expected consequence of sustained antimicrobial selective pressure and the exchange of resistance genes across interconnected human, animal, and environmental sectors in Cambodia.

Taken together, all these findings indicate that AMR in Cambodia involves interacting human, animal, food, and environmental sectors, with healthcare settings acting as important amplification points. However, the evidence is uneven across sectors and provides limited direct evidence of inter-compartment transmission. While the pre-specification of *K. pneumoniae* introduces a potential selection bias, our results nevertheless highlight that it may be a suitable candidate pathogen for sentinel surveillance in healthcare settings within the One Health framework, given its recurrent association with severe infections, MDR and carbapenem resistance, and well-characterized hospital outbreak clusters. Its value as a sentinel should therefore need to be tested in pathogen-neutral, longitudinal, and cross-sectoral surveillance studies.

### 3.4. Risk Factors for AMR Colonization and Infection in Cambodia

The included studies indicate that AMR in Cambodia is associated with a combination of behavioral clinical, environmental, agricultural, and structural risk factors operating simultaneously across different sectors. Supplementary Table S6 shows that the inappropriate use of antibiotics within the human health sector is a major determinant of resistance. This includes self-medication, over-the-counter access and over-prescription by clinicians. For example, some studies highlight a level of inappropriate prescriptions to hospitalized patients of up to 86%. Extensive antimicrobial exposure may favor the survival and expansion of resistant organisms by eliminating susceptible bacteria while allowing ESBL-producing and MDR pathogens, including *K. pneumoniae*, to persist and proliferate. This is called selective pressure [4,6,25]. Other major factors associated with AMR colonization and infection in the country include intensive care unit (ICU) and especially NICU admissions, prolonged hospitalizations and invasive medical procedures. This principally concerns ESBL-producing and carbapenem resistant pathogens, reflecting the major role of hospitals in the amplification of the transmission of AMR pathogens [19,20,21,22,23,53].

Neonatal AMR risk is influenced by maternal colonization; evidence of MDR *K. pneumoniae* has been observed among pregnant women, and there is a potential for partial vertical and/or early life transmission leading to neonatal infection/colonization [38,88]. Moreover, weak immune systems and high exposure to healthcare settings lead to a significant burden of MDR infections in young children and neonates [19,44,70,71]. Adult populations are also at AMR risk because of unprotected sexual activity, limited surveillance, and exposure to STIs; specifically, rising numbers of MDR and XDR strains of *N. gonorrhoeae* have been observed [52,59].

Supplementary Table S6 also highlights structural and environmental factors that play an important role in the dissemination of AMR. In community settings, the lack of proper WASH infrastructure is a major driver of environmental contamination, which in turn fosters AMR colonization and infection [12]. The consumption of contaminated meats, fishes, and fermented products represents another route of AMR transmission, as they have high odds of containing MDR pathogens [15,26,34,58]. Furthermore, zoonotic transmission of ESBL and MDR organisms has also been documented, especially through daily interactions between humans and animals at the household level [10,11,18]. At a larger scale, the use of antibiotics for intensive livestock farming and aquaculture has been identified as an important driver not only of MDR organisms, but also their spread into the food supply [15,16,48,56].

The emergence and spread of AMR pathogens are influenced by socioeconomic barriers to accessing professional medical services [4,6] and structural factors at the health system level, including limited access to diagnostics and limited microbiology surveillance capacity, two factors that can contribute to delays in appropriate treatment initiation [2,3]. Furthermore, inadequate antibiotic regulation at the local and regional levels, together with the presence of substandard and counterfeit antibiotics (i.e., accounting for one third of all antibiotics used in the Southeast Asian region), may contribute to inappropriate antimicrobial exposure and subtherapeutic dosing. These conditions can promote survival pressure [5]. It is important to note that these factors should be interpreted as structural determinants or conditions associated with AMR rather than as independently demonstrated causes of specific transmission events. High population density and shared housing may further facilitate circulation of resistant organisms within communities [25,28,39].

### 3.5. Insights into Klebsiella Pneumoniae in Cambodia

Table 1 provides a summary of the 15 studies included that were related to *K. pneumoniae* in Cambodia. Most of these studies were conducted in hospitals, where within-facility transmission was inferred or demonstrated in outbreak investigations [20,23]. Evidence from genomic studies included further supported inferred nosocomial *K. pneumoniae* transmission, with strains in NICUs exhibiting a high colonization rate (>86%) and well-defined transmission networks within hospitals in general, suggesting substantial amplification of AMR [23]. Furthermore, two articles provided evidence of shared CTX-M producing clones between humans and animals, showing a link between reservoirs in hospital, community, and animal farming sectors [11,18].

Among the 15 studies examining *K. pneumoniae*, reported ESBL prevalence varied across settings, ranging from 30% to 98%. Specifically, in one study, prevalence reached 84% among community-carriage isolates and 98% among hospital-associated isolates [22]. This publication also reported 39% resistance to third-generation cephalosporins. Among bloodstream infection isolates, 98% were ESBL-producing, while an increasing occurrence of carbapenemase-producing *K. pneumoniae* (CRKP) was also reported [27,51]. The latter confers high-level resistance to last-resort carbapenems, significantly limiting treatment options and facilitating the rapid spread of highly resistant strains within both healthcare and community settings. Indeed, approximately 12% of *K. pneumoniae* infections identified in Cambodian hospital settings are carbapenemase-producers, which suggests a rapid emergence of resistance in clinical isolates [24].

*K. pneumoniae* has been reported in community and pediatric populations; however, available estimates are study-specific. One study among children reported that 55% (82/148) of participants carried Enterobacterale isolates containing one or more ESBL genes, including ESBL-producing *K. pneumoniae*; this suggests a substantial burden of ESBL-producing Enterobacterales colonization in the studied population [39]. Another study reported that 82% of *K. pneumoniae* isolates recovered from children were MDR [44], highlighting the potential clinical impact of MDR *K. pneumoniae* infections in this vulnerable population. The high rates of third-generation cephalosporin resistance among *K. pneumoniae* isolates from pediatric infections is a considerable challenge for clinicians when managing pediatric patients [42]. Concerning potential transmission pathways of *K. pneumoniae* among children and newborns, one study reported that 56% of pregnant women carried MDR *K. pneumoniae*, suggesting a potential maternal reservoir and possible risk of neonatal exposure [38]. However, studies investigating *K. pneumoniae* infections in newborns suggest that acquisition occurs more frequently through nosocomial and environmental pathways rather than through confirmed vertical transmission [90]. Moreover, clinical studies show that infections caused by ESBL-producing *K. pneumoniae* strains, particularly pneumonia and bloodstream infections, are associated with increased mortality among adult hospitalized patients [91].

*K. pneumoniae*’s high ESBL production, its MDR profiles, its emerging resistance to carbapenems, its mainly nosocomial transmission pathway, and evidence for its zoonotic circulation, as well as the lack of comprehensive overview about risk factors, are all elements underlining why this organism could be a sentinel, relevant, and representative pathogen for the development of future multidisciplinary research and policy on AMR in general in Cambodia under the One Health approach.

**Table 1:** Evidence of *Klebsiella pneumoniae* AMR in Cambodia (2010–2025) and potential transmission pathways inferred from the available evidence.

Ref.	Study	Year	Setting	Key AMR Finding in *K. pneumoniae*	Population/Sample	One Health Relevance	Potential Transmission Pathway Inferred from the Available Evidence
[24]	Genomic surveillance	2025	Cambodia hospitals	Approximately 12% carbapenemase-producing Enterobacterales, including *K. pneumoniae*, in genomic surveillance	Hospital patients	CRKP emergence	Nosocomial transmission within healthcare setting
[11]	One Health genomic study	2025	Cambodia humans/animals	84% CTX-M prevalence; shared *K. pneumoniae* clones/determinants reported in humans, and animals	Humans/animals	Cross-species transmission	Potential cross-sectoral linkage; direction not established
[90]	Neonatal sources	2025	Cambodia	Non-maternal ESBL Enterobacterales incl. *K. pneumoniae*	Neonates	Environmental/hospital source	Nosocomial/environmental acquisition; direction not established
[50]	National Antimicrobial Resistance Surveillance	2025	National AMR surveillance across 8 sentinel hospitals in Cambodia	*K. pneumoniae* accounted for approximately 16% of reported bacterial isolates in national surveillance, with 39.7% resistance to amoxicillin–clavulanate and 21.6% carbapenem resistance.	38,978 bacterial isolates from mostly blood and cerebrospinal fluid samples	National surveillance highlights hospital and community AMR burden, informing infection control and antimicrobial stewardship strategies	Hospital-associated resistance burden; surveillance does not establish transmission direction
[22]	Cross-sectional laboratory	2025	Phnom Penh hospital	39% third-generation cephalosporin resistance (cross-sectional estimate); ESBL 30%; carbapenemase 4.1%; NDM/OXA-48 detected	Hospital patients	Nosocomial spread and carbapenem resistance emergence	Intra-hospital transmission/emergence; direction not established
[42]	Enterobacterales children	2024	Angkor Hospital for Children, Siem Reap, Cambodia	High 3GC resistance in *K. pneumoniae*	Children	Pediatric burden	Healthcare-associated transmission; direction not established
[38]	Cross-sectional community	2020	Cambodia	56% carriage, MDR common	Pregnant women	Maternal colonization	Community carriage; potential maternal reservoir
[20]	Neonatal transmission	2019	NICU Phnom Penh	MDR transmission linked to antibiotic pressure	Neonates	Hospital amplification	Nosocomial transmission supported by longitudinal genomic evidence
[39]	Cross-sectional colonization	2019	Cambodia children	55% gut ESBL Enterobacterales incl. *K. pneumoniae*	Children	Community colonization	Community colonization
[23]	NICU WGS study	2018	Cambodia NICU	86% colonization with MDR *K. pneumoniae*	Neonates	Genomic transmission clusters	Nosocomial transmission clusters supported by genomic evidence
[44]	Pediatric infections	2018	Cambodia	82% MDR Enterobacterales incl. *K. pneumoniae*	Children	Severe pediatric infections	Healthcare-associated severe infection; pathway not established
[51]	ESBL rise study	2018	Cambodia hospitals	Rapid ESBL expansion in *K. pneumoniae*	Hospital patients	Hospital amplification	Hospital amplification
[92]	Prospective observational study	2018	NICU Phnom Penh	High transmission clusters of *K. pneumoniae*	Neonates	Hospital outbreak dynamics	Nosocomial outbreak dynamics
[27]	Laboratory-based cross-sectional surveillance	2015	Sihanouk Hospital Centre of HOPE, Phnom Penh, Cambodia	98% ESBL CTX-M Enterobacterales incl. *K. pneumoniae*	BSI patients	High hospital burden	Healthcare-associated setting; transmission pathway not established
[91]	Pneumonia study	2010	Cambodia hospital	ESBL *K. pneumoniae* linked to high mortality	Hospital patients	Clinical severity	Nosocomial

## 4. Discussion

AMR represents one of the most pressing global public health threats, particularly concerning Enterobacterales pathogens, which are characterized by rapid acquisition and dissemination of resistance determinants. In Cambodia, multiple bacterial pathogens contribute to the AMR burden, including *K. pneumoniae*, *E. coli*, *Salmonella* spp., *Shigella* spp., *S. pneumoniae*, *M. tuberculosis*, *S. aureus*, *H. pylori*, *C. difficile* and *N. gonorrhoeae*. Each is associated with specific clinical syndromes and resistance profiles. *K. pneumoniae* is clinically important because it causes severe infections, including bloodstream, urinary tract, wound, and respiratory infections, and can acquire resistance determinants such as blaCTX-M, blaNDM, and blaOXA-48 [10,11,22,24,27,86]. While acknowledging that its pre-specification for study inclusion in the present scoping review constitutes a potential selection bias, its repeated association with MDR, carbapenem resistance, and healthcare-associated outbreaks in the selected studies suggests that it could serve as a potential sentinel organism for healthcare-associated AMR surveillance in Cambodia.

*K. pneumoniae* has been principally characterized as a dominant nosocomial pathogen. Documented outbreak clusters and the transmission of genetically related MDR strains highlight that NICUs are major amplification sites [20,23,92]. More generally, *K. pneumoniae* accounts for a significant proportion of the WHO-ESKAPE pathogens present in Cambodia and is the second most diffused pathogen after *E. coli* in national surveillance datasets, representing approximately 16% of reported bacterial isolates [50]. However, compared to *E. coli*, research on *K. pneumoniae* in Cambodia is limited, suggesting that its presence is underestimated. Despite the relevance of investigating *K. pneumoniae* within a One Health framework, available evidence shows that research on this pathogen is particularly fragmented, with limited integration across human, animal, food, and environmental sectors [10,11,18,20,23,24,38,90,92].

Despite growing international interest in AMR, the epidemiology of *K. pneumoniae* in Cambodia remains only partially characterized, particularly outside of healthcare settings. The present scoping review identified major knowledge gaps concerning this pathogen. Most Cambodian studies focus on clinical and neonatal settings, with comparatively limited data on potential transmission dynamics in human–animal–food–environment interfaces [9,10,11,18,93]. Although One Health genomic evidence demonstrates shared ESBL-producing clones between humans and animals—suggesting interspecies transmission—related findings are limited in number and scope [11]. Similarly, evidence of *K. pneumoniae* in food systems, aquaculture, and environmental compartments is sparse, preventing a full understanding of upstream reservoirs and transmission networks [15,16,34,46,48,49,56,58,94]. This creates a critical blind spot in terms of understanding whether hospitals are the primary drivers of persistence or whether repeated external re-introduction contributes substantially to clinical burden [9,26,34].

The Cambodian AMR landscape indicates that *K. pneumoniae* lies at the intersection of multiple transmission pathways involving human–animal–food–environment contamination, but the evidence does not establish a single interconnected transmission system or directional pathway for this pathogen [1,4,5,7,89]. Healthcare exposure, antimicrobial use, agricultural practices, environmental contamination, and structural constraints may create conditions that select for and sustain resistance [4,6,7]. Healthcare-associated risk factors such as prolonged hospitalization, ICU/NICU admission, and invasive procedures further amplify transmission risk [19,20,21,22,23,53,92]. Structural limitations, including weak diagnostic capacity, limited genomic surveillance, inadequate infection prevention and control (IPC), and poor WASH infrastructure, further sustain transmission cycles [2,3,4,12]. Moreover, the widespread use of informal healthcare providers and the presence of substandard and counterfeit antibiotics (reported in approximately 31–32% of surveyed antibiotic samples in some settings) may result in inadequate antimicrobial exposure and promote the selection and spread of AMR bacteria [5]. Agricultural antibiotic use and environmental contamination create a continuous circulation of resistant organisms and resistance genes between humans, animals, and ecosystems [10,11,12,15,16,18]. However, the specific contribution of these non-clinical pathways to *K. pneumoniae* persistence remains poorly characterized [25,28,39]. Clinically, CRKP infections represent a major therapeutic challenge. Treatment options are increasingly limited to older, more toxic, or less effective agents, resulting in prolonged hospitalization, increased healthcare costs, and higher mortality [24,27,51,91]. Vulnerable populations, particularly neonates, young children, and critically ill patients, bear a disproportionate burden due to increased exposure and reduced treatment options [19,20,23,44,92]. As such, the continued expansion of CRKP directly threatens the safety and feasibility of modern medical interventions, including surgery, intensive care, oncology, and transplantation.

This scoping review has several limitations. First, Google Scholar and Google Search, which were used as supplementary discovery sources, are less standardized and less reproducible than conventional bibliographic databases, and their dynamically ranked results may affect retrieval. Second, the English-language restriction, owing to a lack of resources, may have excluded relevant Khmer-language publications, government bulletins, and locally produced surveillance reports. Third, a substantial amount of relevant grey literature may remain unpublished, inaccessible, or insufficiently indexed. This absence from our included texts may translate into an underestimation by this scoping review of the true extent and diversity of AMR in Cambodia. Fourth, our search was oriented toward clinical database coverage; we did not include important sources like Scopus, Embase, CABI Abstracts, or the WHO Western Pacific Region (WPRO) database. This choice may have systematically underrepresented evidence from the animal, food, agricultural, veterinary, and environmental sectors. Fifth, the 94 included studies were highly heterogeneous in design, population, sampling techniques, laboratory methods, and definitions of resistance; this limits direct comparison and precludes quantitative pooling or weighting of prevalence estimates. Sixth, geographic coverage was uneven, and differences in data-collection periods and the lack of standardized longitudinal surveillance limit assessment of temporal trends. Finally, limited genomic data prevented detailed reconstruction of transmission pathways or clonal relationships across sectors. Combined, all these limitations suggest the need for standardized, longitudinal, cross-sectoral AMR surveillance in Cambodia. Although significant advances have been made in implementing national systems and laboratory platforms [50,66,95,96,97], the communication of outbreak-related results is still suboptimal, which hampers tailored AMR policy development [97].

The findings of our scoping review support the need to strengthen Cambodia’s nascent One Health coordination for AMR research, surveillance, and policy development. Priorities should include the expansion of genomic sequencing capacity, environmental and agricultural monitoring, and improved integration of clinical and non-clinical datasets to characterize potential transmission networks. Enhanced infection prevention and control, antimicrobial stewardship, and effective regulation of antibiotic use in both human and veterinary sectors are essential to reduce unnecessary antimicrobial exposure and selective pressure. While acknowledging the caveat of the pre-specification of *K. pneumoniae* in the selection process, our results suggest that this pathogen could be a potential candidate sentinel for healthcare-associated AMR surveillance because of its recurrent association with severe infections, MDR and carbapenem resistance, and documented hospital outbreak clusters. Its broader value as a One Health sentinel needs to be tested through pathogen-neutral, longitudinal, and cross-sectoral surveillance. Sustained investment in genomic epidemiology, intersectoral coordination, and antimicrobial innovation will be essential to address the burden of MDR, colistin, and carbapenem-resistant infections in Cambodia and the wider Southeast Asia region.

## 5. Conclusions

In Cambodia, AMR is a major and heterogeneous public health threat involving human, animal, food, and environmental sectors, although the current evidence base is dominated by clinical and hospital studies. This scoping review highlights widespread MDR and ESBL-producing Enterobacterales, with *K. pneumoniae* frequently implicated in healthcare-associated infections, neonatal outbreaks, and high-priority resistance phenotypes including carbapenem resistance. However, evidence for transmission between sectors remains limited, and reported resistance estimates vary substantially according to population, setting, specimen, sampling period, and laboratory definition. Given these findings, *K. pneumoniae* can best be regarded as a potential sentinel organism for healthcare-associated AMR surveillance rather than as a conclusively established universal One Health sentinel. Strengthening longitudinal genomic surveillance, laboratory capacity, antimicrobial stewardship, infection prevention and control, and integrated One Health monitoring will be critical to characterize potential transmission pathways and guide coordinated AMR actions in Cambodia.

## Figures and Tables

**Figure 1:**
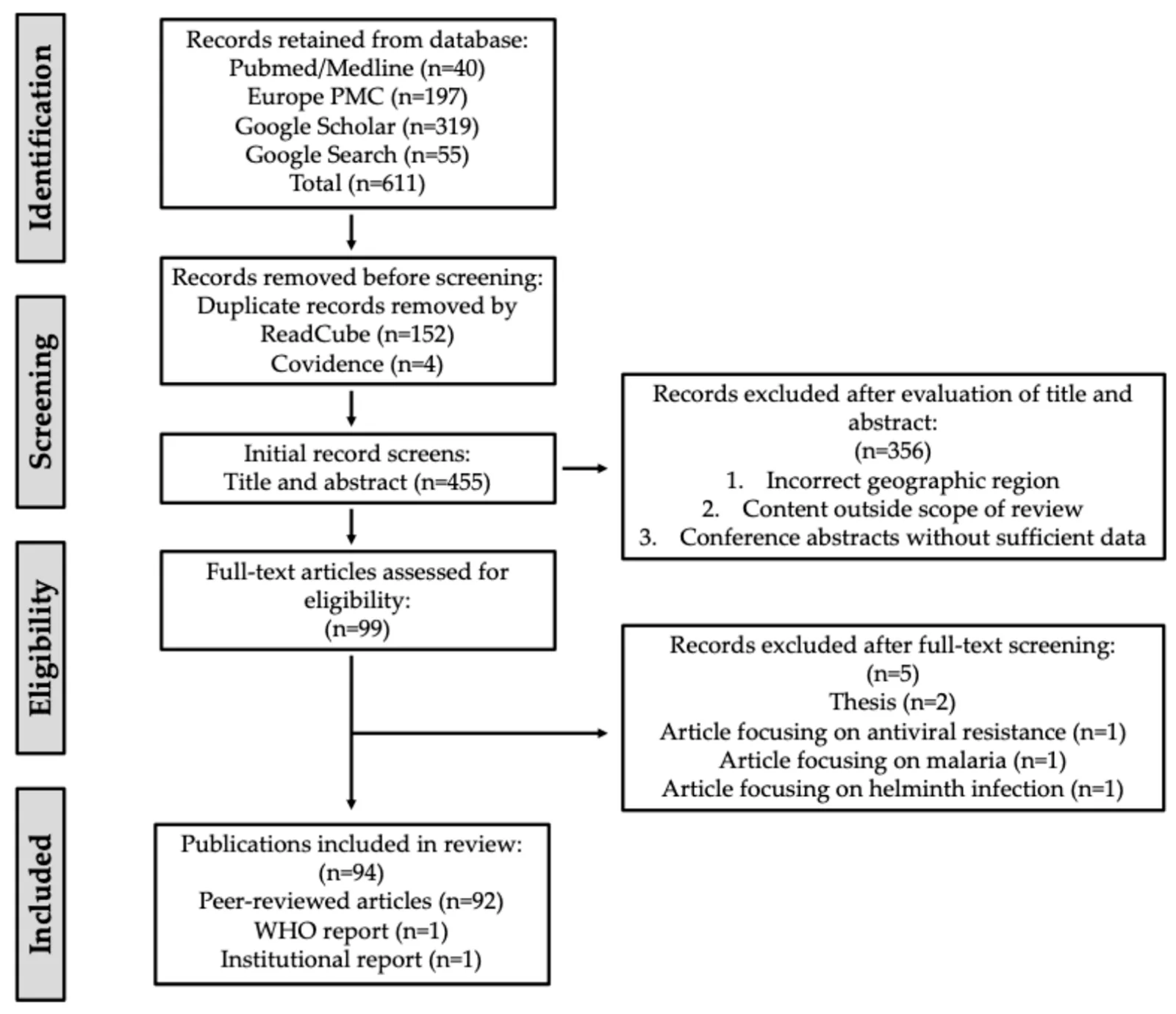
PRISMA-ScR flow diagram detailing records selection process.

## Data Availability

No additional datasets were generated for this scoping review. The data presented in this article were derived from the publicly available published literature.

## Supplementary Materials

The following supporting information can be downloaded at: https://www.mdpi.com/article/10.3390/microorganisms14102161/s1, Table S1: Database-specific search strategies; Table S2: Inclusion and exclusion criteria for AMR surveillance research in Cambodia included in the scoping review; Table S3: Core Evidence Map of AMR Studies in Cambodia using a One Health framework; Table S4: Antimicrobial Resistance Patterns by Pathogen in Cambodia-based Studies; Table S5: Map of interconnected AMR transmission pathways in Cambodia; Table S6: Risk Factors Associated with AMR Colonization and Infection in Cambodia.

## Abbreviations

The following abbreviations are used in this manuscript.

Abbreviation	Definition
3GC	Third-generation cephalosporins
AMR	Antimicrobial resistance
AmpC	AmpC β-lactamase
blaCTX-M	Gene encoding cefotaximase-Munich β-lactamase
blaNDM	Gene encoding New Delhi metallo-β-lactamase
blaOXA-48	Gene encoding oxacillinase-48 carbapenemase
BSI	Bloodstream infection
CRKP	Carbapenem-resistant *Klebsiella pneumoniae*
CTX-M	Cefotaximase-Munich β-lactamase
ESBL	Extended-spectrum β-lactamase
ESKAPE	*Enterococcus faecium*, *Staphylococcus aureus*, *Klebsiella pneumoniae*, *Acinetobacter baumannii*, *Pseudomonas aeruginosa*, and *Enterobacter* spp.
FC428	Internationally disseminated multidrug-resistant *Neisseria gonorrhoeae* clone
gyrA	DNA gyrase subunit A gene
H58	Multidrug-resistant *Salmonella Typhi* H58 lineage
ICU	Intensive care unit
MDR	Multidrug-resistant
MDR-TB	Multidrug-resistant tuberculosis
mecA	Methicillin resistance gene
mcr	Mobilized colistin resistance gene family
mphA	Macrolide phosphotransferase gene A
MRSA	Methicillin-resistant *Staphylococcus aureus*
NDM	New Delhi metallo-β-lactamase
NICU	Neonatal intensive care unit
NTS	Non-typhoidal *Salmonella*
OH4CAM	One Health for Cambodia
OXA-48	Oxacillinase-48 carbapenemase
PBPs	Penicillin-binding proteins
PDR	Pan-drug-resistant
penA	Penicillin-binding protein 2 gene
rpoB	RNA polymerase β-subunit gene
STI	Sexually transmitted infection
TB	Tuberculosis
UTI	Urinary tract infection
VRE	Vancomycin-resistant *Enterococcus*
VRSA	Vancomycin-resistant *Staphylococcus aureus*
WASH	Water, sanitation, and hygiene
WGS	Whole-genome sequencing
XDR	Extensively drug-resistant
23S rRNA	23S ribosomal RNA

## References

[1] Chea B., Kong S., Thim S., Ban N., Seng S., Fernandez-Colorado C., Kang K. (2022). Knowledge, Attitudes, and Practices of Antimicrobial Use and Resistance among Livestock Producers in Cambodia. Open J. Anim. Sci..

[2] Getchell M., Wulandari S., de Alwis R., Agoramurthy S., Khoo Y.K., Mak T.-M., Moe L., Stona A.-C., Pang J., Momin M.H.F.H.A. (2024). Pathogen Genomic Surveillance Status among Lower Resource Settings in Asia. Nat. Microbiol..

[3] Mao S., Soputhy C., Lay S., Jacobs J., Ku G.M., Chau D., Chhea C., Ir P. (2023). The Barriers and Facilitators of Implementing a National Laboratory-Based AMR Surveillance System in Cambodia: Key Informants’ Perspectives and Assessments of Mi-crobiology Laboratories. Front. Public Health.

[4] Om C., Daily F., Vlieghe E., McLaughlin J.C., McLaws M.-L. (2017). Pervasive Antibiotic Misuse in the Cambodian Community: Antibiotic-Seeking Behaviour with Unrestricted Access. Antimicrob. Resist. Infect. Control.

[5] Yong Y.L., Plançon A., Lau Y.H., Hostetler D.M., Fernández F.M., Green M.D., Sounvoravong S., Nara S., Boravann M., Dumrong T. (2014). Collaborative Health and Enforcement Operations on the Quality of Antimalarials and Antibiotics in Southeast Asia. Am. J. Trop. Med. Hyg..

[6] Om C., Daily F., Vlieghe E., McLaughlin J.C., McLaws M.-L. (2016). “If It’s a Broad Spectrum, It Can Shoot Better”: Inappropriate Antibiotic Prescribing in Cambodia. Antimicrob. Resist. Infect. Control.

[7] Om C., Vlieghe E., McLaughlin J.C., Daily F., McLaws M.-L. (2016). Antibiotic Prescribing Practices: A National Survey of Cambodian Physicians. Am. J. Infect. Control.

[8] Murray C.J.L., Ikuta K.S., Sharara F., Swetschinski L., Aguilar G.R., Gray A., Han C., Bisignano C., Rao P., Wool E. (2022). Global Burden of Bacterial Antimicrobial Resistance in 2019: A Systematic Analysis. Lancet.

[9] Nadimpalli M., Vuthy Y., de Lauzanne A., Fabre L., Criscuolo A., Gouali M., Huynh B.-T., Naas T., Phe T., Borand L. (2019). Meat and Fish as Sources of Extended-Spectrum β-Lactamase–Producing *Escherichia coli*, Cambodia. Emerg. Infect. Dis..

[10] Nadimpalli M.L., Stegger M., Viau R., Yith V., de Lauzanne A., Sem N., Borand L., Huynh B.-T., Brisse S., Passet V. (2023). Plugging the Leaks: Antibiotic Resistance at Human-Animal Interfaces in Low-Resource Settings. Front. Ecol. Environ..

[11] Elmarghani E.D., Pettersson J.H.-O., Atterby C., Hickman R.A., Seng S., San S., Osbjer K., Magnusson U., Mourkas E., Järhult J.D. (2025). Genomic Insights into Extended-Spectrum β-Lactamase- and Plasmid-Borne AmpC-Producing *Escherichia coli* Transmission between Humans and Livestock in Rural Cambodia. J. Med. Microbiol..

[12] Yoneda I., Rozanah U.N., Nishiyama M., Mith H., Watanabe T. (2022). Detection and Genetic Analysis of *Escherichia coli* from Tonle Sap Lake and Its Tributaries in Cambodia: Spatial Distribution, Seasonal Variation, Pathogenicity, and Antimicrobial Resistance. Environ. Pollut..

[13] Getchell M., Wulandari S., de Alwis R., Agoramurthy S., Khoo Y.K., Mak T.-M., Pang J., Moe L., Stona A.-C., Lim J.C. (2023). Pathogen Genomic Surveillance in Asia: A Multi-Country Assessment. Preprint.

[14] Ahn Y., Jin S., Park G., Lee H.Y., Lee H., Shin E., Kim J., Yoo J., Kim Y. (2024). Epidemiological Analysis and Prevention Strategies in Response to a Shigellosis Cluster Outbreak: A Retrospective Case Series in an Alternative School in the Republic of Korea. Osong Public Health Res. Perspect..

[15] Seyha D., Rithy C., Samnang N., Trevanich S., NoraNavarro-Gonzalez, Calvez S., Rortana C., Vutey V. (2024). Antimicrobial Resistance of *Aeromonas* spp. and *Vibrio* spp. Isolated from Fresh Pangasius Fish in Cambodia. J. Environ. Sci. Eng. B.

[16] Peng C., Moniroth S., Khy P., Chea S., Thanh C., Heng O., Sarter S., Cheng S., Caruso D. (2023). Antibiotic Resistance Profiles of Sentinel Bacteria Isolated from Aquaculture in Cambodia. J. Water Health.

[17] Gysin M., Hon P.Y., Tan P., Sengduangphachanh A., Simmalavong M., Hinfonthong P., Kaewphanderm N., Pham T.D., Nguyen T.H., Haldimann K. (2022). Apramycin Susceptibility of Multidrug-Resistant Gram-Negative Blood Culture Isolates in Five Countries in Southeast Asia. Int. J. Antimicrob. Agents.

[18] Atterby C., Osbjer K., Tepper V., Rajala E., Hernandez J., Seng S., Holl D., Bonnedahl J., Börjesson S., Magnusson U. (2019). Carriage of Carbapenemase- and Extended-Spectrum Cephalosporinase-Producing *Escherichia coli* and Klebsiella Pneumoniae in Humans and Livestock in Rural Cambodia; Gender and Age Differences and Detection of blaOXA-48 in Humans. Zoonoses Public Health.

[19] Turner P., Pol S., Soeng S., Sar P., Neou L., Chea P., Day N.P., Cooper B.S., Turner C. (2016). High Prevalence of Antimicrobial-Resistant Gram-Negative Colonization in Hospitalized Cambodian Infants. Pediatr. Infect. Dis. J..

[20] Crellen T., Turner P., Pol S., Baker S., Nguyen T.N.T., Stoesser N., Day N.P., Turner C., Cooper B.S. (2019). Transmission Dynamics and Control of Multidrug-Resistant Klebsiella Pneumoniae in Neonates in a Developing Country. eLife.

[21] Nov V., Chau D., Chhea C. (2020). High Rates of Drug Resistance in *Escherichia coli* from a Pilot Antimicrobial Resistance Surveillance System in Cambodia. Open Forum Infect. Dis..

[22] Delvallez G., Cheng S., Girond F., Krang S., Meng S., Nop P., Heng S., Han S., Keo S., Kong K. (2025). Prevalence and Characterization of Third-Generation Cephalosporin, Carbapenem and Colistin-Resistant Enterobacterales Isolated from Clinical Samples in Cambodia. N. Microbes N. Infect..

[23] Smit P.W., Stoesser N., Pol S., van Kleef E., Oonsivilai M., Tan P., Neou L., Turner C., Turner P., Cooper B.S. (2018). Trans-mission Dynamics of Hyper-Endemic Multi-Drug Resistant Klebsiella Pneumoniae in a Southeast Asian Neonatal Unit: A Longitudinal Study with Whole Genome Sequencing. Front. Microbiol..

[24] Yek C., Lon C., Chea S., Lay S., Oum M.H., Tang G., Lon C., Pacheco A.R., Drobish I., Stuehser R. (2025). Bacterial Genomics for National Antimicrobial Resistance Surveillance in Cambodia. J. Infect. Dis..

[25] de Lauzanne A., Sreng N., Foucaud E., Sok T., Chon T., Yem C., Hak V., Heng S., Soda M., Gouali M. (2022). Prevalence and Factors Associated with Faecal Carriage of Extended-Spectrum β-Lactamase-Producing Enterobacterales among Peri-partum Women in the Community in Cambodia. J. Antimicrob. Chemother..

[26] Chrun R., Mith H., Meng S., Long S., Born P., Inatsu Y. (2023). Assessing Prevalence and Antibiotic Resistance of *Escherichia coli* and Other Enterobacteriaceae Isolated from Cambodian Fermented Fish and Vegetables. Jpn. Agric. Res. Q. JARQ.

[27] Vlieghe E.R., Huang T.-D., Phe T., Bogaerts P., Berhin C., Smet B.D., Peetermans W.E., Jacobs J.A., Glupczynski Y. (2015). Prevalence and Distribution of Beta-Lactamase Coding Genes in Third-Generation Cephalosporin-Resistant Enterobacteriaceae from Bloodstream Infections in Cambodia. Eur. J. Clin. Microbiol. Infect. Dis..

[28] Singh S.R., Mao B., Evdokimov K., Tan P., Leab P., Ong R., Vonthanak S., Tam C.C., Hsu L.Y., Turner P. (2020). Prevalence of MDR Organism (MDRO) Carriage in Children and Their Household Members in Siem Reap Province, Cambodia. JAC Antimicrob. Resist..

[29] Thanh D.P., Thompson C.N., Rabaa M.A., Sona S., Sopheary S., Kumar V., Moore C., Thieu N.T.V., Wijedoru L., Holt K.E. (2016). The Molecular and Spatial Epidemiology of Typhoid Fever in Rural Cambodia. PLoS Neglected Trop. Dis..

[30] The H.C., Rabaa M.A., Thanh D.P., Lappe N.D., Cormican M., Valcanis M., Howden B.P., Wangchuk S., Bodhidatta L., Mason C.J. (2016). South Asia as a Reservoir for the Global Spread of Ciprofloxacin-Resistant Shigella Sonnei: A Cross-Sectional Study. PLoS Med..

[31] Poramathikul K., Wojnarski M., Sok S., Sokh V., Chiek S., Seng H., Krang S., Ly S., Nou S., Chann S. (2021). Update on Shigella and Nontyphoidal Salmonella Antimicrobial Drug Resistance: Implications on Empirical Treatment of Acute Infectious Diarrhea in Cambodia. Antimicrob. Agents Chemother..

[32] Kasper M.R., Sokhal B., Blair P.J., Wierzba T.F., Putnam S.D. (2010). Emergence of Multidrug-Resistant *Salmonella enterica* Serovar Typhi with Reduced Susceptibility to Fluoroquinolones in Cambodia. Diagn. Microbiol. Infect. Dis..

[33] Emary K., Moore C.E., Chanpheaktra N., An K.P., Chheng K., Sona S., Duy P.T., Nga T.V.T., Wuthiekanun V., Amornchai P. (2012). Enteric Fever in Cambodian Children Is Dominated by Multidrug-Resistant H58 *Salmonella enterica* Serovar Typhi with Intermediate Susceptibility to Ciprofloxacin. Trans. R. Soc. Trop. Med. Hyg..

[34] Schwan C.L., Lomonaco S., Bastos L.M., Cook P.W., Maher J., Trinetta V., Bhullar M., Phebus R.K., Gragg S., Kastner J. (2021). Genotypic and Phenotypic Characterization of Antimicrobial Resistance Profiles in Non-Typhoidal *Salmonella enterica* Strains Isolated From Cambodian Informal Markets. Front. Microbiol..

[35] Moore C.E., Sona S., Poda S., Putchhat H., Kumar V., Sopheary S., Stoesser N., Bousfield R., Day N., Parry C.M. (2016). Antimicrobial Susceptibility of Uropathogens Isolated from Cambodian Children. Paediatr. Int. Child Health.

[36] Stoesser N., Sheppard A.E., Moore C.E., Golubchik T., Parry C.M., Nget P., Saroeun M., Day N.P.J., Giess A., Johnson J.R. (2015). Extensive Within-Host Diversity in Fecally Carried Extended-Spectrum-Beta-Lactamase-Producing *Escherichia coli* Isolates: Implications for Transmission Analyses. J. Clin. Microbiol..

[37] Phe T., Vlieghe E., Reid T., Harries A.D., Lim K., Thai S., Smet B.D., Veng C., Kham C., Ieng S. (2013). Does HIV Status Affect the Aetiology, Bacterial Resistance Patterns and Recommended Empiric Antibiotic Treatment in Adult Patients with Bloodstream Infection in Cambodia?. Trop. Med. Int. Health.

[38] Huynh B.-T., Passet V., Rakotondrasoa A., Diallo T., Kerleguer A., Hennart M., Lauzanne A.D., Herindrainy P., Seck A., Bercion R. (2020). Klebsiella Pneumoniae Carriage in Low-Income Countries: Antimicrobial Resistance, Genomic Diversity and Risk Factors. Gut Microbes.

[39] Van Aartsen J.J., Moore C.E., Parry C.M., Turner P., Phot N., Mao S., Suy K., Davies T., Giess A., Sheppard A.E. (2019). Epidemiology of Paediatric Gastrointestinal Colonisation by Extended Spectrum Cephalosporin-Resistant *Escherichia coli* and Klebsiella Pneumoniae Isolates in North-West Cambodia. BMC Microbiol..

[40] Hide M., Meng S., Cheng S., Bañuls A.-L., Ky S., Yay C., Laurent D., Delvallez G. (2024). Colistin Resistance in ESBL- and Carbapenemase-Producing *Escherichia coli* and Klebsiella Pneumoniae Clinical Isolates in Cambodia. J. Glob. Antimicrob. Resist..

[41] Singh S.R., Tang C.Y., Mao B., Soeng S., Ling C.L., Teo J.Q.-M., Vonthanak S., Turner P., Hsu L.Y., Ong R.T.-H. (2023). Whole Genome Sequencing of Multidrug Resistant Enterobacterales Identified in Children and Their Household Members within Siem Reap, Cambodia. JAC Antimicrob. Resist..

[42] Ardura-Garcia C., Bran S., Sar P., Suy K., Pol S., Lee S.J., Ling C.L., Corander J., Turner P. (2026). Antibiotic and Healthcare Exposure Impact on Dynamics of Third-Generation Cephalosporin-Resistant Enterobacterales Colonisation in Cambodian Children: A Six-Month Cohort Study. Antimicrob. Resist. Infect. Control.

[43] Ruppé E., Hem S., Lath S., Gautier V., Ariey F., Sarthou J.-L., Monchy D., Arlet G. (2009). CTX-M β-Lactamases in *Escherichia coli* from Community-Acquired Urinary Tract Infections, Cambodia. Emerg. Infect. Dis..

[44] Fox-Lewis A., Takata J., Miliya T., Lubell Y., Soeng S., Sar P., Rith K., McKellar G., Wuthiekanun V., McGonagle E. (2018). Antimicrobial Resistance in Invasive Bacterial Infections in Hospitalized Children, Cambodia, 2007-Emerg. Infect. Dis..

[45] Vlieghe E.R., Phe T., Smet B.D., Veng H.C., Kham C., Lim K., Koole O., Lynen L., Peetermans W.E., Jacobs J.A. (2013). Bloodstream Infection among Adults in Phnom Penh, Cambodia: Key Pathogens and Resistance Patterns. PLoS ONE.

[46] Lay K.K., Jeamsripong S., Sunn K.P., Angkititrakul S., Prathan R., Srisanga S., Chuanchuen R. (2021). Colistin Resistance and ESBL Production in Salmonella and *Escherichia coli* from Pigs and Pork in the Thailand, Cambodia, Lao PDR, and Myanmar Border Area. Antibiotics.

[47] Trongjit S., Angkittitrakul S., Chuanchuen R. (2016). Occurrence and Molecular Characteristics of Antimicrobial Resistance of *Escherichia coli* from Broilers, Pigs and Meat Products in Thailand and Cambodia Provinces. Microbiol. Immunol..

[48] Ström G., Boqvist S., Albihn A., Fernström L.-L., Djurfeldt A.A., Sokerya S., Sothyra T., Magnusson U. (2018). Antimicrobials in Small-Scale Urban Pig Farming in a Lower Middle-Income Country—Arbitrary Use and High Resistance Levels. Antimicrob. Resist. Infect. Control.

[49] Pungpian C., Lee S., Trongjit S., Sinwat N., Angkititrakul S., Prathan R., Srisanga S., Chuanchuen R. (2021). Colistin Resistance and Plasmid-Mediated Mcr Genes in *Escherichia coli* and Salmonella Isolated from Pigs, Pig Carcass and Pork in Thailand, Lao PDR and Cambodia Border Provinces. J. Vet. Sci..

[50] Mot V., Chau D., Sopheab H. (2025). The Bacterial Profiles and Antimicrobial Susceptibility Patterns of Isolates from National Antimicrobial Resistance Surveillance, Cambodia 2018–2021. Cambodia J. Public Health.

[51] Caron Y., Chheang R., Puthea N., Soda M., Boyer S., Tarantola A., Kerléguer A. (2018). Beta-Lactam Resistance among Enterobacteriaceae in Cambodia: The Four-Year Itch. Int. J. Infect. Dis..

[52] Ouk V., Pham C.D., Wi T., van Hal S.J., Lahra M.M. (2023). The Enhanced Gonococcal Surveillance Programme, Cambodia. Lancet Infect. Dis..

[53] Taitt C.R., Leski T.A., Heang V., Ford G.W., Prouty M.G., Newell S.W., Vora G.J. (2015). Antimicrobial Resistance Genotypes and Phenotypes from Multidrug-Resistant Bacterial Wound Infection Isolates in Cambodia. J. Glob. Antimicrob. Resist..

[54] Kuijpers L.M.F., Phe T., Veng C.H., Lim K., Ieng S., Kham C., Fawal N., Fabre L., Hello S.L., Vlieghe E. (2017). The Clinical and Microbiological Characteristics of Enteric Fever in Cambodia, 2008–2015. PLoS Neglected Trop. Dis..

[55] Kietsiri P., Sornsakrin S., Nou S., Oransathid W., Peerapongpaisarn D., Oransathid W., Nobthai P., Wassanarungroj P., Gonwong S., Sakpaisal P. (2025). Understanding the Etiology of Diarrheal Illness in Cambodia in a Case-Control Study from 2020 to 2023. Gut Pathog..

[56] Chea B., Kong S., Thim S., Ban N., Chrun R., Venn V., Fernandez-Colorado C., Kang K. (2025). Prevalence and Antimicrobial Resistance of *Salmonella* spp. Isolated from Swine and Poultry Farms in Cambodia. Vet. World.

[57] Maubaret C., Caméléna F., Mrimèche M., Braille A., Liberge M., Mainardis M., Guillaume C., Noel F., Bébéar C., Molina J.-M. (2023). Two Cases of Extensively Drug-Resistant (XDR) Neisseria Gonorrhoeae Infection Combining Ceftriax-one-Resistance and High-Level Azithromycin Resistance, France, November 2022 and May 2023. Eurosurveillance.

[58] Huoy L., Nasirzadeh L., Phan K., Tieng S., Sternberg-Lewerin S., Bongcam-Rudloff E., Boqvist S. (2025). Antimicrobial Resistance and Serotype Distribution of *Salmonella* spp. Isolated from Fresh Foods in Cambodia. J. Appl. Microbiol..

[59] Maatouk I., Vumbugwa P., Cherdtrakulkiat T., Heng L.S., Hoffman I., Palaypayon N., Kakooza F., Kittiyaowamarn R., Kyambadde P., Lahra M.M. (2025). Antimicrobial Resistance in Neisseria Gonorrhoeae in Nine Sentinel Countries within the World Health Organization Enhanced Gonococcal Antimicrobial Surveillance Programme (EGASP), 2023: A Retrospective Observational Study. Lancet Reg. Health West. Pac..

[60] Poncin T., Merimeche M., Braille A., Mainardis M., Bebear C., Jacquier H., Berçot B. (2019). Two Cases of Multidrug-Resistant Neisseria Gonorrhoeae Related to Travel in South-Eastern Asia, France, June 2019. Eurosurveillance.

[61] Vlieghe E.R., Phe T., Smet B.D., Veng C.H., Kham C., Bertrand S., Vanhoof R., Lynen L., Peetermans W.E., Jacobs J.A. (2012). Azithromycin and Ciprofloxacin Resistance in Salmonella Bloodstream Infections in Cambodian Adults. PLoS Neglected Trop. Dis..

[62] Hout B., Oum C., Men P., Vanny V., Supaprom C., Heang V., Rachmat A., Prouty M., Newell S., Harrison D. (2015). Drug Resistance in Bacteria Isolated from Patients Presenting with Wounds at a Non-Profit Surgical Center in Phnom Penh, Cambodia from 2011–2013. Trop. Dis. Travel Med. Vaccines.

[63] Trongjit S., Angkititrakul S., Tuttle R.E., Poungseree J., Padungtod P., Chuanchuen R. (2017). Prevalence and Antimicrobial Resistance in *Salmonella enterica* Isolated from Broiler Chickens, Pigs and Meat Products in Thailand–Cambodia Border Provinces. Microbiol. Immunol..

[64] Kuijpers L.M.F., Hello S.L., Fawal N., Fabre L., Tourdjman M., Dufour M., Sar D., Kham C., Phe T., Vlieghe E. (2016). Genomic Analysis of *Salmonella enterica* Serotype Paratyphi A during an Outbreak in Cambodia, 2013–2015. Microb. Genom..

[65] One Health Initiative (2026). Cambodia Launches One Health Project to Strengthen Health System and Outbreak Response. https://onehealthinitiative.com/cambodia-launches-one-health-project-to-strengthen-health-system-and-outbreak-response/.

[66] World Health Organization (2025). Multi-Sectoral Action Plan on Antimicrobial Resistance in Cambodia 2025–2029. https://cdn.who.int/media/docs/default-source/antimicrobial-resistance/amr-spc-npm/nap-library/amr_msap_cambodia_2025-2029.pdf?sfvrsn=3f6b5dd_1&download=true.

[67] Tricco A.C., Lillie E., Zarin W., O’Brien K.K., Colquhoun H., Levac D., Moher D., Peters M.D.J., Horsley T., Weeks L. (2018). PRISMA Extension for Scoping Reviews (PRISMA-ScR): Checklist and Explanation. Ann. Intern. Med..

[68] Hirabayashi A., Yanagisawa H., Takahashi H., Yahara K., Boeing P., Wolfenden B., Nov V., Lorn V., Veng M., Ann V. (2021). On-Site Genomic Epidemiological Analysis of Antimicrobial-Resistant Bacteria in Cambodia with Portable Laboratory Equipment. Front. Microbiol..

[69] Nadimpalli M., Fabre L., Yith V., Sem N., Gouali M., Delarocque-Astagneau E., Sreng N., Hello S.L., the BIRDY study group (2019). CTX-M-55-Type ESBL-Producing *Salmonella enterica* Are Emerging among Retail Meats in Phnom Penh, Cambodia. J. Antimicrob. Chemother..

[70] Huynh B.-T., Kermorvant-Duchemin E., Chheang R., Randrianirina F., Seck A., Ratsima E.H., Andrianirina Z.Z., Diouf J.-B., Abdou A.Y., Goyet S. (2021). Severe Bacterial Neonatal Infections in Madagascar, Senegal, and Cambodia: A Multicentric Community-Based Cohort Study. PLoS Med..

[71] Meng C.Y., Smith B.L., Bodhidatta L., Richard S.A., Vansith K., Thy B., Srijan A., Serichantalergs O., Mason C.J. (2011). Etiology of Diarrhea in Young Children and Patterns of Antibiotic Resistance in Cambodia. Pediatr. Infect. Dis. J..

[72] Dusadeepong R., Delvallez G., Cheng S., Meng S., Sreng N., Letchford J., Choun K., Teav S., Hardy L., Jacobs J. (2023). Phylogenomic Investigation of an Outbreak of Fluoroquinolone-Resistant *Salmonella enterica* subsp. Enterica Serovar Paratyphi A in Phnom Penh, Cambodia. Microb. Genom..

[73] Kheng C., Meas V., Pen S., Sar P., Turner P. (2020). Salmonella Typhi and Paratyphi A Infections in Cambodian Children, 2012–2016. Int. J. Infect. Dis..

[74] Poramathikul K., Bodhidatta L., Chiek S., Oransathid W., Ruekit S., Nobthai P., Lurchachaiwong W., Serichantalergs O., Lon C., Swierczewski B. (2016). Multidrug-resistant Shigella infections in patients with diarrhea, Cambodia, 2014–2015. Emerg. Infect. Dis..

[75] Na S., Intanon M., Srithanasuwan A., Chaisri W., Suriyasathaporn W. (2025). Evidence of Vancomycin-Resistant Staphylococcus Aureus, Multidrug-Resistant S. Aureus, and Enterococcus Faecium-Causing Mastitis in Thailand and Cambodia. Vet. World.

[76] Antony S.J., Faix D., Gollogly J.G., June K.Y., Oy H., Savuth C., Chonthida S., Putnam S.D. (2020). A Retrospective Observational Study of Traumatic Orthopaedic: Related Infections in Cambodia. Int. J. Res. Orthop..

[77] Nickerson E.K., Wuthiekanun V., Kumar V., Amornchai P., Wongdeethai N., Chheng K., Chantratita N., Putchhat H., Thaipadungpanit J., Day N.P. (2011). Emergence of Community-Associated Methicillin-Resistant Staphylococcus Aureus Carriage in Children in Cambodia. Am. J. Trop. Med. Hyg..

[78] Belman S., Soeng S., Soputhy C., Gladstone R., Hawkins P.A., Breiman R.F., McGee L., Bentley S.D., Lo S.W., Turner P. (2022). Genetic Background of Cambodian Pneumococcal Carriage Isolates Following Pneumococcal Conjugate Vaccine 13. Microb. Genom..

[79] Moore C.E., Giess A., Soeng S., Sar P., Kumar V., Nhoung P., Bousfield R., Turner P., Stoesser N., Day N.P.J. (2016). Characterisation of Invasive Streptococcus Pneumoniae Isolated from Cambodian Children between 2007–2012. PLoS ONE.

[80] Surcouf C., Heng S., Pierre-Audigier C., Cadet-Daniel V., Namouchi A., Murray A., Gicquel B., Guillard B. (2011). Molecular Detection of Fluoroquinolone-Resistance in Multi-Drug Resistant Tuberculosis in Cambodia Suggests Low Association with XDR Phenotypes. BMC Infect. Dis..

[81] Edokimov K., Yamada Y., Dary C., Miow Q.H., Hsu L.-Y., Ong R.T.-H., Saphonn V. (2022). Whole-Genome Sequencing of Mycobacterium Tuberculosis from Cambodia. Sci. Rep..

[82] Aumpan N., Vilaichone R.-K., Gumnarai P., Sanglutong L., Ratanachu-ek T., Mahachai V., Yamaoka Y. (2020). Prevalence and Antibiotic Resistance Patterns of Helicobacter Pylori Infection in Koh Kong, Cambodia. Asian Pac. J. Cancer Prev. APJCP.

[83] Tuan V.P., Narith D., Tshibangu-Kabamba E., Dung H.D.Q., Viet P.T., Sokomoth S., Binh T.T., Sokhem S., Tri T.D., Ngov S. (2019). A Next-Generation Sequencing-Based Approach to Identify Genetic Determinants of Antibiotic Resistance in Cambodian Helicobacter Pylori Clinical Isolates. J. Clin. Med..

[84] Eng L., Collins D.A., Alene K.A., Bory S., Theng Y., Vann P., Meng S., Limsreng S., Clements A.C.A., Riley T.V. (2025). Clostridioides (Clostridium) Difficile Infection in Hospitalized Adult Patients in Cambodia. Microbiol. Spectr..

[85] Eng L., Putsathit P., Lim S.-C., Chisholm J.M., Collins D.A., Clements A.C.A., Alene K.A., Riley T.V. (2025). Antimicrobial Resistance of Clostridioides (Clostridium) Difficile in Cambodia. Antibiotics.

[86] Marcy E., Chiek S., Hidé M., Hak S., Ma C., Lem M., Delvallez G., Bañuls A.-L., Cheng S., Hayer J. (2025). Hybrid Sequencing of Chromosome and Plasmids from Multidrug-Resistant *Escherichia coli* Isolated in Cambodia: Are Megaplasmids Vectors of Antibiotic Resistance Genes?. J. Glob. Antimicrob. Resist..

[87] Beaumont A.-L., de Lauzanne A., Criscuolo A., Fabre L., Rabenandrasana M.A.N., Randriamanga N.F., Bernabeu S., Harimanana A., Randremanana R.V., Herindrainy P. (2025). Contribution of Maternal Gut Carriage to Neonatal Acquisition of Extended-Spectrum Beta-Lactamase-Producing Enterobacterales in Madagascar and Cambodia. Nat. Commun..

[88] Chomkatekaew C., Thaipadungpanit J., Hearn P., Soeng S., Pol S., Neou L., Hopkins J., Turner P., Batty E.M. (2023). Detection of Maternal Transmission of Resistant Gram-Negative Bacteria in a Cambodian Hospital Setting. Front. Microbiol..

[89] Om C., McLaws M.-L. (2016). Antibiotics: Practice and Opinions of Cambodian Commercial Farmers, Animal Feed Retailers and Veterinarians. Antimicrob. Resist. Infect. Control.

[90] Beaumont A.-L., de Lauzanne A., Criscuolo A., Fabre L., Rabenandrasana M., Randriamanga N., Bernabeu S., Ha-rimanana A., Randremanana R., Herindrainy P. (2025). Early Neonatal Acquisition of Extended-Spectrum Beta-Lactamase (ESBL)-Producing Enterobacterales in Madagascar and Cambodia: Clonal and Plasmid-Level Contributions of Maternal Gut Carriage. Preprint.

[91] Rammaert B., Goyet S., Beauté J., Hem S., Te V., Try P.L., Mayaud C., Borand L., Buchy P., Guillard B. (2012). Klebsiella Pneumoniae Related Community-Acquired Acute Lower Respiratory Infections in Cambodia: Clinical Characteristics and Treatment. BMC Infect. Dis..

[92] Crellen T., Turner P., Pol S., Baker S., Nguyen T.N.T., Stoesser N., Day N.P.J., Cooper B.S. (2018). Transmission Dynamics and Between-Species Interactions of Multidrug-Resistant Enterobacteriaceae. bioRxiv.

[93] Auguet O.T., Niehus R., Gweon H.S., Berkley J.A., Waichungo J., Njim T., Edgeworth J.D., Batra R., Chau K., Swann J. (2021). Population-Level Faecal Metagenomic Profiling as a Tool to Predict Antimicrobial Resistance in Enterobacterales Isolates Causing Invasive Infections: An Exploratory Study across Cambodia, Kenya, and the UK. eClinicalMedicine.

[94] Shahum A., Sladeckova V., Benca J., Krcmery V., Sternnberg N., Dudova Z., Liskova A., Mikloskova M., Bencova M., Hoin H.L.H.C. (2017). Reversibility of Antibiotic Resistance in an Orphanage of Children with AIDS in Cambodia. Clin. Soc. Work Health Interv..

[95] Oonsivilai M., Mo Y., Luangasanatip N., Lubell Y., Miliya T., Tan P., Loeuk L., Turner P., Cooper B.S. (2018). Using Machine Learning to Guide Targeted and Locally-Tailored Empiric Antibiotic Prescribing in a Children’s Hospital in Cambodia. Wellcome Open Res..

[96] Nov V., Chau D., Pa K., Hem K., Krang S. (2020). Proficiency Testing Performances Analysis of Microbiology Laboratories Participating in Cambodia Antimicrobial Resistance (AMR) Surveillance System. Infect. Control Hosp. Epidemiol..

[97] Wynberg E., Mishra A., Liverani M., Vanna M., Chanpheakdey P., Chaikul K., Suy K., Bory S., Voeurng B., Callery J.J. (2026). Impact of an Electronic Clinical Decision Support Algorithm (eCDSA) on Antibiotic Prescribing in Primary Care in Cambodia: A Cluster Randomised Controlled Trial. Int. J. Infect. Dis..

