# Supplementary material for: A One Health Scoping Review of Antimicrobial Resistance in Cambodia: Evidence Mapping and Insights into Klebsiella pneumoniae
Source: Microorganisms. 2026 Sep 24;14(10):2161. doi: 10.3390/microorganisms14102161 (PMC13707576; doi:10.3390/microorganisms14102161)
Supplement: Supplementary file 1 [file microorganisms-14-02161-s001.zip › Supplementary/Supplementary Table S1.pdf]

Supplementary Table S1. Database-specific search strategies

The following search concepts and Boolean combinations were used to identify evidence on antimicrobial resistance (AMR) in Cambodia across the human, animal, food, and environmental sectors within a One Health framework, with a pre-specified focus on *Klebsiella pneumoniae*. Search syntax was adapted to the indexing system and search functionality of each source. PubMed/MEDLINE and Europe PMC were used as the primary bibliographic databases, whereas Google Scholar and Google Search were used as supplementary discovery sources to identify potentially relevant grey literature, non-indexed evidence, and other sources that might not be retrieved through conventional bibliographic databases.

**Source**	**Search strategy / search concepts**	**Limitation/ notes**
**PubMed/MEDLINE**	("Antimicrobial Resistance"[Mesh] OR "Drug Resistance, Bacterial"[Mesh] OR "antimicrobial resistance" OR "antibiotic resistance" OR MDR OR XDR OR PDR OR ESBL OR "carbapenem resistance") AND (Cambodia[Mesh] OR Cambodia OR Cambodian) AND ("Klebsiella pneumoniae" OR Enterobacterales OR ESKAPE OR "Escherichia coli" OR Salmonella OR Shigella OR "Staphylococcus aureus" OR "Neisseria gonorrhoeae" OR "Streptococcus pneumoniae" OR "Mycobacterium tuberculosis" OR "Helicobacter pylori" OR "Clostridioides difficile")	Search conducted through 11 May 2026. English-language publications only; no restriction by publication year. The initial search yielded 203 records. Following an initial relevance assessment, 40 records were retained for formal screening.
**Europe PMC**	("antimicrobial resistance" OR "antibiotic resistance" OR MDR OR XDR OR PDR OR ESBL OR "carbapenem resistance") AND (Cambodia OR Cambodian) AND ("Klebsiella pneumoniae" OR Enterobacterales OR ESKAPE OR "Escherichia coli" OR Salmonella OR Shigella OR "Staphylococcus aureus" OR "Neisseria gonorrhoeae" OR "Streptococcus pneumoniae" OR "Mycobacterium tuberculosis" OR "Helicobacter pylori" OR "Clostridioides difficile")	The initial search yielded 1,447 records. Following screening for relevance and eligibility, 197 records were retained for the formal screening dataset.

**Google Scholar**	"antimicrobial resistance" OR "antibiotic resistance" Cambodia AND ("Klebsiella pneumoniae" OR Enterobacterales OR ESKAPE OR "Escherichia coli" OR Salmonella OR Shigella OR "Staphylococcus aureus" OR ESBL OR carbapenem)	Supplementary discovery source used to identify potentially relevant peer-reviewed and grey literature. Because Google Scholar results are dynamically ranked and are not equivalent to conventional bibliographic database yields, results were screened sequentially for relevance to Cambodia and the eligibility criteria. The initial search yielded 8,030 records, of which 319 were retained for inclusion in the formal screening dataset.
**Google Search**	(Cambodia OR Cambodian) AND ("antimicrobial resistance" OR "antibiotic resistance" OR ESBL OR "carbapenem resistance") AND (Klebsiella OR Enterobacterales OR "E. coli" OR Salmonella OR Shigella OR MRSA)	Supplementary discovery source used to identify potentially relevant government, institutional, surveillance, and other grey-literature sources. 55 records were identified and retained for formal eligibility assessment after sequential screening for relevance.
**Government and institutional sources**	Targeted searches of relevant Cambodian government agencies, public-health institutions, research institutions, and institutional repositories for reports, surveillance documents, and other grey literature addressing AMR in human, animal, food, and environmental sectors.	Used to identify grey literature and non-indexed evidence potentially relevant to the review. Potentially eligible records were retained for duplicate removal and formal eligibility assessment.

Abbreviations: AMR, antimicrobial resistance; ESBL, extended-spectrum β-lactamase; ESKAPE, *Enterococcus faecium*, *Staphylococcus aureus*, *Klebsiella pneumoniae*, *Acinetobacter baumannii*, *Pseudomonas aeruginosa*, and *Enterobacter* spp.; MDR, multidrug resistance; MRSA, methicillin-resistant *Staphylococcus aureus*; PDR, pan-drug resistance; XDR, extensively drug-resistant.
