# Supplementary material for: A One Health Scoping Review of Antimicrobial Resistance in Cambodia: Evidence Mapping and Insights into Klebsiella pneumoniae
Source: Microorganisms. 2026 Sep 24;14(10):2161. doi: 10.3390/microorganisms14102161 (PMC13707576; doi:10.3390/microorganisms14102161)
Supplement: Supplementary file 1 [file microorganisms-14-02161-s001.zip › Supplementary/Supplementary Table S2.pdf]

Supplementary Table S2. Inclusion and exclusion criteria for AMR surveillance research in Cambodia included in the scoping review.

Criteria	Inclusion	Exclusion
**Population/Setting**	Studies conducted in Cambodia involving the following sectors: environment, animal, or human populations in hospital and/or in community healthcare settings	Studies not conducted in Cambodia
**Concepts**	Antimicrobial resistance (AMR), including antibiotic resistance, multidrug resistance (MDR), extensive drug-resistance (XDR), pan-drug resistance (PDR), carbapenem resistance, and ESBL-production	Studies not addressing AMR; studies focusing on non-antibacterial resistance (e.g., antiviral, antifungal, antiparasitic resistance)
**Pathogens**	Bacterial pathogens, with emphasis on ESKAPE organisms (*Enterococcus faecium* [VRE], *Staphylococcus aureus* [MRSA], *Klebsiella pneumoniae*, *Acinetobacter baumannii*, *Pseudomonas aeruginosa*, and *Enterobacter spp.*), as well as other Enterobacterales	Studies exclusively focusing on non-bacterial organisms (e.g., viruses, fungi, parasites)
**Outcomes**	Epidemiological measures (prevalence, incidence), resistance patterns, clinical outcomes (e.g., mortality, treatment failure), and drivers/risk factors (e.g., antibiotic use, prescribing practices, healthcare factors)	Studies not reporting AMR-related outcomes or lacking primary data (e.g., purely methodological or laboratory validation studies without epidemiological or clinical findings)
**Study design**	Observational studies (cross-sectional, cohort, case-control), surveillance reports, laboratory-based studies, and public health or global health reports	Editorials, commentaries, opinion pieces, conference abstracts without sufficient data, or studies lacking methodological detail
**Time frame**	No restriction on publication year	Not applicable
**Language**	Articles published in English;	Non-English language publications
**Publication type**	Peer-reviewed articles and relevant grey literature (e.g., government and institutional reports)	Non-credible sources or documents lacking sufficient methodological rigor
