# Supplementary material for: A One Health Scoping Review of Antimicrobial Resistance in Cambodia: Evidence Mapping and Insights into Klebsiella pneumoniae
Source: Microorganisms. 2026 Sep 24;14(10):2161. doi: 10.3390/microorganisms14102161 (PMC13707576; doi:10.3390/microorganisms14102161)
Supplement: Supplementary file 1 [file microorganisms-14-02161-s001.zip › Supplementary/Supplementary Table S3.pdf]

Supplementary Table S3. Core Evidence Map of AMR Studies in Cambodia using a One Health framework.

Domain	Humans	Animals	Food	Environment	Neonates / Children	Clinical Relevance
K. pneumoniae	[18–20,23,24, 38–45]	[18,24,68]	[10,18,38]	[18,24,68]	[19,20,23,39,42,44]	ESBL, MDR, carbapenem resistance, and emerging colistin resistance; predominantly reported in healthcare settings, including bloodstream infections, and NICU outbreaks
E. coli	[20–22,24,25, 27,35–45]	[46–49]	[26,34,46,49,58]	[9–12,18]	[19,35,39,42,44,70,74]	ESBL (CTX-M dominant), MDR, plasmid-mediated AmpC, emerging carbapenem and colistin resistance; dominant Enterobacterales in community, food chain, and clinical infections
**Salmonella spp.**	[45,61]	[56,63]	[34,56,58,63]	None	[29,32,33,54,64,71,72]	Typhoidal and non-typhoidal Salmonella; foodborne transmission; pediatric burden
**Shigella spp.**	[30,31,73]	None	None	None	[31,73]	High AMR in diarrheal disease; major pediatric pathogen
Neisseria gonorrhoeae	[52,57,59, 60]	None	None	None	None	Increasing resistance to first-line antibiotics; STI concern
***Staphylococcus aureus* (MRSA)**	[53,62,75]	[76]	None	[28]	[77]	MRSA in hospitals and community; zoonotic interface potential
Streptococcus pneumoniae	[78,79]	None	None	None	[78,79]	Respiratory pathogen; pediatric infections; vaccine-preventable AMR burden
Mycobacterium tuberculosis	[80,81]	None	None	None	None	Drug-resistant TB; major public health burden in Cambodia
Helicobacter pylori	[82,83]	None	None	None	None	Gastric pathogen; emerging resistance to clarithromycin/metronidazole
Clostridioides difficile	[84,85]	None	None	None	None	Healthcare-associated infection; antibiotic-associated colitis
**Aquatic bacteria (Aeromonas, Vibrio)**	None	[16]	[15]	[16]	None	Aquatic AMR reservoirs; environmental and food chain relevance
**Mixed Enterobacterales**	[9,10]	[11]	[9,34]	[11,12,18]	[28]	Other One Health/cross-domain AMR

Note: Reference lists overlap where included studies reported multiple pathogens; each pathogen-specific entry was cross-checked against the original study. Evidence of co-occurrence or shared determinants does not by itself establish directional transmission.
