# Supplementary material for: A One Health Scoping Review of Antimicrobial Resistance in Cambodia: Evidence Mapping and Insights into Klebsiella pneumoniae
Source: Microorganisms. 2026 Sep 24;14(10):2161. doi: 10.3390/microorganisms14102161 (PMC13707576; doi:10.3390/microorganisms14102161)
Supplement: Supplementary file 1 [file microorganisms-14-02161-s001.zip › Supplementary/Supplementary Table S4.pdf]

Supplementary Table S4. Antimicrobial Resistance Patterns by Pathogen in Cambodia-based Studies.

Pathogen	Key Resistance Patterns	Dominant Mechanisms / Genes	Settings / Populations	Study References
K. pneumoniae	High ESBL rates; MDR and carbapenem-resistant strains reported in hospitals; colistin resistance reported in some clinical isolates	ESBLs (CTX-M); carbapenemases (e.g., OXA-48 and others); plasmid-mediated resistance; clonal expansion in hospitals; colistin resistance mechanisms	Neonatal units; pediatric wards; hospital bloodstream infections; community carriage (children, households); limited livestock overlap	[9–12,18,20–22,24,25,35–48]
E. coli	High ESBL prevalence; MDR in community, food, and clinical isolates; carbapenem and colistin resistance reported in certain settings	CTX-M ESBLs (especially CTX-M types); plasmid-mediated AmpC; carbapenemases (e.g., OXA-48); mcr genes (colistin resistance); high plasmid exchange	Humans (community, peripartum women, children, neonates); livestock; food (meat, fish, fermented foods); environment (rivers/lakes); hospitals	[9–12,18,20–22,24,25,35–48]
**Salmonella spp. (Typhi, Paratyphi, NTS)**	High MDR (50 to 91%); fluoroquinolone resistance; ceftriaxone resistance increasing	H58 lineage (Typhi), plasmid-mediated MDR	Enteric fever patients, children, poultry, food markets	[29,32–34,45,54,56,58,61,63,64,71,72]
**Shigella spp.**	Extremely high MDR (up to 91%); ciprofloxacin resistance common	gyrA, mphA mutations/genes	Diarrhea, mainly in children	[30,31,73]
Neisseria gonorrhoeae	MDR and XDR strains; reduced susceptibility and resistance to ceftriaxone	FC428 lineage; mosaic penA mutations	STI clinics, adults	[52,57,59,60]
***Staphylococcus aureus* (MRSA/VRSA)**	High MRSA prevalence (up to 61%); rare vancomycin resistance (VRSA)	mecA; van genes (rare)	Wound infections, hospitals, livestock (mastitis), community	[28,53,62,75–77]
Streptococcus pneumoniae	Moderate MDR; increasing penicillin resistance; serotype replacement post-vaccine	Altered PBPs	Pediatric respiratory infections	[78,79]
Mycobacterium tuberculosis	Overall low MDR-TB; emerging fluoroquinolone resistance	rpoB, gyrA mutations	TB patients	[80,81]
Helicobacter pylori	High MDR (57 to 76%); strong	23S rRNA mutations	Adults (gastric infection)	[82,83]

	metronidazole resistance	(clarithromycin), others		
Clostridioides difficile	Notable colonization (<24%); emerging resistance patterns	Toxin-associated strains; limited AMR gene data	Hospitalized adults	[84,85]
**Aquatic bacteria (Aeromonas spp., Vibrio spp.)**	MDR common in aquaculture and fish markets	Diverse mobile resistance elements	Fish, aquatic environments, food markets	[15,16]
