# Supplementary material for: A One Health Scoping Review of Antimicrobial Resistance in Cambodia: Evidence Mapping and Insights into Klebsiella pneumoniae
Source: Microorganisms. 2026 Sep 24;14(10):2161. doi: 10.3390/microorganisms14102161 (PMC13707576; doi:10.3390/microorganisms14102161)
Supplement: Supplementary file 1 [file microorganisms-14-02161-s001.zip › Supplementary/Supplementary Table S5.pdf]

**Supplementary Table S5.** Map of interconnected AMR transmission pathways in Cambodia.

| AMR organism/pathogen | Source sectors involved | Potential transmission pathways | Key evidence (Cambodia studies) | Evidence type | One Health implication |
|---|---|---|---|---|---|
| ***K. pneumoniae* (MDR, ESBL)** | Humans (hospital) ⇔ Environment (hospital surfaces) | Potential healthcare-based transmission, antibiotic selection pressure | NICU outbreaks; high transmission clusters; antibiotic-driven spread[20,23] | Genomic + clinical | Hospitals are key reservoirs; infection control critical |
| **ESBL-producing *E. coli* (CTX-M)** | Humans ⇔ Livestock ⇔ Environment | Direct contact, fecal contamination, shared water/soil; potential cross-sectoral linkage | Shared CTX-M genes and clones across humans and animals; 84% CTX-M prevalence; household spread [10,11,18,41] | Genomic + epidemiological | Evidence consistent with cross-sectoral linkage; integrated surveillance is needed |
| **MDR *E. coli* (including mcr genes)** | Humans ⇔ Livestock ⇔ Food chain | Potential food-chain exposure; antimicrobial use in farms | mcr-1/3 detected in livestock and food; MDR prevalence up to 79% in farms [46,48,49] | Molecular + observational | Potential food-chain pathway; livestock antimicrobial use may contribute to the selection of antimicrobial-resistant Enterobacterales. |
| **ESBL Enterobacterales (general)** | Humans ⇔ Hospitals ⇔ Community | Healthcare-associated and community circulation; direction not established | 79% ESBL carriage in community; hospital ESBL rates up to 98% [24,25,27] | Surveillance + clinical | Food systems may represent potential AMR exposure pathways |
| **Salmonella spp. (MDR)** | Humans ⇔ Livestock ⇔ Food markets | Potential food-chain pathway; slaughterhouse contamination | 69–91% MDR in animals and food; linked to human infections [56,58,63] | One Health + food surveillance | Food systems are major AMR transmission routes |
| **Shigella spp. (MDR)** | Humans ⇔ Community ⇔ Environment | Person-to-person, water-associated exposure | 91% MDR; resistant lineages spreading regionally [30,31,73] | Epidemiological + genomic | Poor sanitation accelerates AMR spread |
| ***Salmonella Typhi* (MDR H58 lineage)** | Humans ⇔ Environment (water/sanitation) | Fecal–oral exposure via contaminated water | 85% MDR H58 lineage dominant; widespread in children [29,32,33] | Genomic + epidemiological | Water and sanitation are critical control points |
| ***Staphylococcus aureus* (MRSA, VRSA)** | Humans ⇔ Livestock | Direct contact and occupational exposure; possible zoonotic linkage | MRSA common in hospitals; VRSA detected in dairy cattle [62,75,76] | Clinical + veterinary | Zoonotic transmission risk emerging |
| ***Neisseria gonorrhoeae* (MDR/XDR)** | Humans ⇔ Global transmission networks | Sexual transmission and international spread | XDR strains including FC428 lineage; ceftriaxone resistance [52,57,59] | Surveillance + genomic | International spread influences local AMR burden |
| **Enteric bacteria (mixed MDR)** | Humans ⇔ Food ⇔ Environment | Food contamination and potential | >50% MDR in fermented food; food-human transmission | Food safety + epidemiology | Food hygiene is a major intervention point |

| | | | | | |
|---|---|---|---|---|---|
| | | food-chain exposure | documented [9,26,34] | | |
| **Aquatic MDR bacteria (Aeromonas spp., Vibrio spp.)** | Environment ⇔ Food (fish) ⇔ Humans | Aquaculture antimicrobial use and water-associated exposure | MDR aquatic pathogens in fish markets and farms [15,16] | Environmental + food surveillance | Aquatic systems act as AMR reservoirs |
| **AMR genes (plasmid-mediated)** | Humans ⇔ Animals ⇔ Environment | Horizontal gene transfer via plasmids; gene-level dissemination rather than directional transmission | Megaplasmids and ESBL gene transfer across sectors [10,11,86] | Genomic | Gene-level transmission accelerates AMR spread |
| **Community AMR carriage (ESBL, MRSA)** | Humans ⇔ Households ⇔ Animals | Household contact and shared environment | High ESBL/MRSA carriage in families and communities [25,28,39] | Epidemiological | Community reservoirs sustain AMR circulation |
| **Neonatal AMR pathogens (ESBL Enterobacterales)** | Hospital ⇔ Environment (non-maternal) | Healthcare-associated acquisition; environmental source possible | Neonatal infections not primarily maternal; hospital/environment source [87,88] | Clinical + genomic | Infection control more critical than maternal screening |
| **Antibiotic use practices (drivers)** | Humans ⇔ Livestock ⇔ Health system | Antimicrobial use and prescribing practices as selection pressures | High misuse in farmers and clinicians; weak regulation [1,4,7,89] | Behavioral + policy | Behavioral drivers underpin all transmission pathways |
