# Supplementary material for: A One Health Scoping Review of Antimicrobial Resistance in Cambodia: Evidence Mapping and Insights into Klebsiella pneumoniae
Source: Microorganisms. 2026 Sep 24;14(10):2161. doi: 10.3390/microorganisms14102161 (PMC13707576; doi:10.3390/microorganisms14102161)
Supplement: Supplementary file 1 [file microorganisms-14-02161-s001.zip › Supplementary/Supplementary Table S6.pdf]

Supplementary Table S6. Risk Factors Associated with AMR Colonization and Infection in Cambodia.

Risk factor category	Specific risk factor	Population (s) affected	Key evidence (Cambodia studies)	AMR outcome	Evidence type	One Health relevance (risk/structural determinant)	K. pneumoniae explicitly documented?
**Antibiotic use (human)**	Self-medication, over-the-counter access	General community	High levels of self-medication and inappropriate antibiotic use [4,6,7]	Increased ESBL and MDR carriage	Behavioral + epidemiological	Drives community AMR reservoir	NO
**Antibiotic prescribing practices**	Overprescription by clinicians	Hospital patients	Up to 86% inappropriate prescribing reported [7]	Selection of MDR pathogens	Clinical + behavioral	Amplifies hospital AMR burden	NO
**Livestock antibiotic use**	Prophylactic and growth-promoting antibiotic use	Farmers, rural households	High misuse in livestock systems [1,89]	Emergence of MDR and mcr genes	Observational + One Health	Links animal and human AMR	NO
**Hospital exposure**	ICU/NICU admission	Neonates, critically ill patients	High MDR transmission in NICUs; ESBL and carbapenem resistance common [19,20,23]	Colonization and infection with MDR organisms	Clinical + genomic	Hospitals act as AMR amplification hubs	YES
**Invasive medical care**	Devices, prolonged hospitalization	Hospital patients	High rates of resistant bloodstream infections and wound infections [21,22,53]	ESBL, carbapenemase infections	Clinical	Potential maternal/environmental exposure; direction not established	YES
**Maternal carriage**	Colonisation during pregnancy	Neonates, pregnant women	High MDR carriage in pregnant women; partial maternal-neonate transmission [38,88]	Neonatal colonization	Clinical + molecular	Vertical + environmental transmission	NO
**Neonatal colonization**	Vertical/environmental transmission	Neonates	High MDR colonization in NICUs [15,16]	Severe neonatal infections	Clinical	Early-life AMR risk	YES
**Environmental sanitation**	Poor water, sanitation, hygiene (WASH)	Rural and peri-urban populations	AMR bacteria detected in water	Community colonization with MDR organisms	Environmental + epidemiological	Environment as reservoir	NO

			systems [12]				
**Food exposure**	Consumption of contaminated food (meat, fish, fermented foods)	General population	MDR bacteria in food (>50% in some products) [15,26,34,58]	Foodborne AMR transmission	Food safety studies	Potential food-chain exposure pathway	NO
**Close human–animal contact**	Household livestock rearing	Rural households	Shared AMR genes between humans and animals [10,11,18]	ESBL and MDR colonization	Genomic + One Health	Potential cross-sectoral linkage	NO
**Community crowding**	Household clustering, shared living spaces	Families, children	High ESBL and MRSA carriage in households [25,28,39]	Sustained AMR circulation	Epidemiological	Community-level amplification	NO
**Age (young children)**	Immature immunity, high exposure	Children	High MDR burden in pediatric infections [44,74]	Increased infection risk	Clinical	Vulnerable population group	YES
**Neonatal vulnerability**	Immature immune system, hospital exposure	Neonates	High resistance to first-line antibiotics [19,70]	Severe MDR infections	Clinical	High-risk group	YES
**Sexual behavior & STI exposure**	Unprotected sex, limited surveillance	Adults	Rising MDR/XDR *N. gonorrhoeae* [52,59]	Drug-resistant STI infections	Surveillance	Human-to-human transmission	NO
**Drug quality**	Substandard or falsified antibiotics	General population	Up to 31–32% substandard drugs in region [5]	Subtherapeutic dosing → resistance	Market surveillance	Structural driver of AMR	NO
**Diagnostic limitations**	Limited microbiology capacity	National health system	Weak AMR lab coverage and surveillance gaps [3]	Delayed/incorrect treatment	Health systems research	System-level driver	NO
**Socioeconomic factors**	Limited access to healthcare, informal treatment	Rural populations	Reliance on informal providers and antibiotics [4,6]	Increased misuse and resistance	Behavioral + policy	Underlying structural driver	NO
**Agricultural practices**	Intensive farming, poor biosecurity	Farmers, food systems	High MDR prevalence in poultry and livestock [48,56]	Spread of resistant bacteria	Observational	Links environment, food, and humans	NO

**Aquaculture exposure**	Antibiotic use in fish farming	Consumers, workers	MDR aquatic pathogens detected [15,16]	Environmental AMR exposure	Environmental	Expands AMR reservoir	NO

Note: Factors listed here are risk factors or structural determinants associated with AMR. They are not, by themselves, evidence of confirmed transmission pathways.
